# Interfacial Bond Behavior and Load-Transfer Characteristics of CFRP-Strengthened Traditional Masonry with Glutinous Rice Mortar

**DOI:** 10.3390/ma19183823

**Published:** 2026-09-08

**Authors:** Xiao Liu, Yilun Li, Chaoyang Liu, Haiwei Yao, Liangyin Huang

**Affiliations:** 1 School of Civil Engineering and Architecture, Southwest University of Science and Technology, Mianyang 621010, China; 17639823070@163.com (Y.L.); liuchaoyang0822@163.com (C.L.); 13668353680@163.com (H.Y.); huang13778164203@163.com (L.H.); 2 Shock and Vibration of Engineering Materials and Structures Key Laboratory of Sichuan Province, Mianyang 621010, China

**Keywords:** carbon fiber-reinforced polymer (CFRP), traditional masonry, glutinous rice mortar, masonry interface, interfacial bond behavior, finite element analysis

## Abstract

**Highlights:**

- Interfacial bond behavior of CFRP-strengthened glutinous rice mortar masonry was characterized.
- CFRP bond width significantly affects the load-transfer behavior of CFRP–masonry interfaces.
- Interface debonding reduces the load-carrying capacity and changes the bond response.
- Numerical analysis revealed stress redistribution within the CFRP–masonry interface.
- The findings support performance evaluation of CFRP strengthening for traditional masonry interfaces.

**Abstract:**

Traditional brick masonry buildings in China are commonly constructed using fired clay grey bricks bonded with glutinous rice mortar, forming a unique historical masonry system with significant cultural value. During long-term service, these structures are vulnerable to environmental deterioration, material aging, and seismic actions, resulting in cracking, deformation, and degradation of structural integrity and load-carrying capacity. Carbon fiber-reinforced polymer (CFRP) sheets have been increasingly applied for strengthening masonry structures due to their high strength-to-weight ratio, corrosion resistance, and convenient installation. However, most existing studies on Fiber-reinforced polymer (FRP)–masonry interfaces have focused on conventional masonry systems, while the interfacial bond behavior and load-transfer characteristics between CFRP sheets and traditional grey brick masonry bonded with glutinous rice mortar remain insufficiently investigated. This study investigates the interfacial bond behavior of CFRP-strengthened traditional grey brick masonry through combined experimental testing and numerical analysis. First, uniaxial compression tests were conducted to determine the mechanical properties of glutinous rice mortar and fired clay grey bricks. Subsequently, double-shear tests considering different CFRP bond widths, bond lengths, and interface integrity conditions were performed to characterize the failure modes, force–displacement responses, and interfacial load-carrying behavior. The effects of interface geometric and integrity conditions were considered to evaluate the load-transfer characteristics of the strengthened interface. Based on the experimental results, a finite element model considering interface behavior was established and verified through comparison with the experimental results, which was subsequently employed to investigate the influence of bond width on interfacial stress transfer behavior beyond the experimental conditions. The results show that interfacial debonding accompanied by near-surface masonry damage dominates the failure process of CFRP–glutinous rice mortar masonry interfaces. Increasing the CFRP bond width enhances the interfacial load-carrying capacity and initial stiffness, while the ultimate capacity exhibits an approximately linear relationship with bond width within the investigated range. Numerical analyses further demonstrate that increasing bond width expands the effective load-transfer region, redistributes interfacial stresses, and delays stiffness degradation. These findings improve the understanding of interfacial bond behavior and load-transfer characteristics in CFRP-strengthened traditional masonry systems and provide references for the design and performance evaluation of strengthening applications in historic masonry structures.

## 1. Introduction

Traditional brick masonry buildings in China are commonly constructed using fired clay grey bricks bonded with glutinous rice mortar, forming a unique masonry system with significant historical and cultural value. However, long-term environmental deterioration, material degradation, and seismic actions may induce cracking, surface weathering, deformation, and local instability, thereby compromising the structural integrity and load-bearing capacity of these historic structures. Previous seismic investigations have demonstrated that masonry-based historic structures are vulnerable to severe damage under earthquake loading [1,2].

Fiber-reinforced polymer (FRP) composites have been increasingly applied for strengthening masonry structures due to their high strength-to-weight ratio, corrosion resistance, and convenient installation, showing great potential for the conservation of historic masonry buildings [3,4]. Among various FRP materials, carbon fiber-reinforced polymer (CFRP) sheets are widely used owing to their high elastic modulus, excellent tensile strength, and favorable deformation compatibility [5,6,7]. However, the effectiveness of CFRP strengthening systems is largely governed by the interfacial bond behavior between CFRP sheets and masonry substrates. Interfacial debonding often occurs before the tensile capacity of FRP sheets is fully mobilized and represents a critical failure mode of FRP-strengthened masonry structures [8,9]. Compared with concrete, masonry is a heterogeneous composite material composed of bricks, mortar, and brick–mortar interfaces, resulting in complex stress transfer and crack propagation mechanisms at FRP–masonry interfaces. Moreover, traditional glutinous rice mortar differs significantly from modern cement-based mortars in composition, microstructure, and mechanical properties, leading to uncertainties regarding its interfacial bond behavior. Recent studies have also shown that bio-additives can influence the morphology and performance of lime-based mortars [10]. Compared with conventional cement-based mortar, glutinous rice mortar generally exhibits lower stiffness and strength but relatively better deformation compatibility with traditional fired clay grey bricks, which may affect the stress transfer and debonding behavior of the CFRP–masonry interface. Therefore, understanding the bond behavior between CFRP sheets and grey brick masonry bonded with glutinous rice mortar is essential for developing reliable strengthening methods for traditional masonry structures.

Extensive studies have investigated the interfacial behavior of FRP-strengthened concrete and masonry structures. For FRP–concrete interfaces, Chajes et al. [11] characterized the interfacial stress transfer mechanism through single-shear tests. With the application of FRP strengthening to masonry substrates, various experimental methods, including single-shear and double-shear tests, have been adopted to evaluate load–slip responses, failure modes, effective bond length, and interfacial stress distribution. Among them, double-shear tests have been widely used because of their symmetric loading configuration and reduced eccentricity effects. Valluzzi et al. [12] compared different shear test configurations and provided comprehensive experimental data regarding the bond behavior of FRP-strengthened masonry interfaces.

European researchers have also made important contributions to the strengthening of masonry structures using externally bonded composite systems. For example, brick masonry vaults strengthened with FRP laminates have been investigated by Valluzzi, Valdemarca, and Modena, showing that the strengthening effectiveness of FRP systems is closely related to the bond behavior and failure mechanism of the masonry–reinforcement system [13]. In addition to organic-matrix FRP systems, mortar-based composite systems have been widely investigated in Europe. De Felice et al. reviewed mortar-based systems for externally bonded strengthening of masonry and emphasized the importance of reinforcement–masonry compatibility, substrate properties, and interfacial behavior in strengthened masonry structures [14]. Moreover, studies on ceramic and clay brick masonry walls have shown that reinforcement arrangement can influence the strength and deformability of masonry walls. Jasiński investigated the influence of bed-joint reinforcement on the strength and deformability of masonry shear walls, indicating that masonry-unit characteristics and reinforcement configuration are important factors affecting wall behavior [15].

Although CFRP/epoxy systems have been widely used for masonry strengthening, their performance may be affected by temperature because of the organic polymer matrix. For masonry strengthening applications where thermal performance and material compatibility are of particular concern, inorganic composite systems, such as fabric-reinforced cementitious matrix (FRCM) or inorganic-matrix composite systems, have also been investigated as alternative strengthening solutions [14,16].

The influence of bond geometry parameters, particularly bond width and bond length, has also attracted considerable attention. Chen and Teng [17] developed anchorage strength models for FRP and steel plates bonded to concrete and investigated factors affecting interfacial capacity. Rotunno et al. [18] demonstrated that bonded area significantly influences the ultimate load, load–displacement response, and failure modes of CFRP-strengthened masonry specimens. Chen and Teng [19] further provided a comprehensive collection of studies on FRP bond behavior, including the effects of material properties and bond characteristics. In addition to FRP properties and geometric parameters, masonry substrate characteristics play an important role in interfacial behavior. Aiello and Sciolti [20] and Mazzotti and Murgo [21] highlighted the influence of mortar joints on interfacial stress transfer and debonding mechanisms. Carloni and Subramaniam [22,23] further revealed the influence of mortar joints on stress distribution during debonding. Ghiassi et al. [24] demonstrated the importance of considering mortar joints and masonry heterogeneity in FRP-strengthened masonry analysis, while Faella et al. [25] investigated the influence of masonry characteristics on FRP bond behavior. Jin et al. [26] studied the bond behavior of CFRP–clay brick interfaces using double-shear tests and obtained corresponding load–slip responses and failure characteristics. Nevertheless, most existing studies have focused on conventional clay bricks, cement-based mortar masonry, or stone masonry, while the interfacial behavior of traditional grey brick masonry bonded with glutinous rice mortar remains insufficiently understood.

Numerical investigations of FRP interfaces have mainly focused on bond–slip relationships, debonding prediction, and damage evolution mechanisms. Lu et al. [8] proposed bond–slip models for FRP–concrete interfaces based on experimental and numerical studies, while Yao et al. [27] investigated the applicability of bond–slip models for predicting debonding behavior. With the extension of numerical analyses to masonry structures, Ceroni et al. [9], Grande and Imbimbo [28], and Ghiassi et al. [24] highlighted the importance of considering mortar joints and masonry heterogeneity. Interface-based numerical approaches, including cohesive zone models, have also been adopted to describe interfacial damage evolution and debonding processes. Chen et al. [29] employed cohesive elements with a bilinear constitutive relationship to simulate FRP–masonry interface behavior, and Su et al. [30] demonstrated that combining homogenized masonry models with interface elements can provide a reasonable balance between computational efficiency and simulation accuracy.

Overall, previous studies have established valuable experimental and numerical approaches for investigating FRP-strengthened conventional masonry systems. These studies have clarified important aspects such as test methods, failure modes, bond–slip behavior, effective bond length, stress-transfer mechanisms, and numerical simulation strategies. However, most existing bond-strength and bond–slip models have been developed or calibrated based on conventional masonry substrates, such as clay brick, stone, or cement-/lime-based masonry. For traditional grey brick masonry bonded with glutinous rice mortar, experimental evidence on CFRP–masonry interfacial bond behavior remains limited. In particular, the effects of bond geometry and local interface defects on the force–displacement response, peak load-carrying capacity, failure mode, and stress-transfer characteristics of this traditional material system are still insufficiently understood. Therefore, further experimental characterizations and experimental–numerical comparisons are needed to clarify the CFRP–masonry load-transfer characteristics of traditional grey brick masonry bonded with glutinous rice mortar.

Therefore, this study investigates the interfacial bond behavior and load-transfer characteristics of CFRP sheets bonded to grey brick masonry with glutinous rice mortar through combined experimental and numerical approaches. Different from most previous studies on CFRP-strengthened conventional masonry, this study focuses on a traditional grey brick masonry system bonded with glutinous rice mortar and examines the effects of bond length, bond width, and local interface defects on the CFRP–masonry load-transfer behavior. Double-shear tests were conducted to characterize the failure modes, force–displacement responses, and interfacial load-carrying behavior of CFRP-strengthened traditional masonry, with bond length and interface debonding ratio considered as additional interface conditions. Subsequently, a finite element model considering interface behavior was established and compared with the experimental results. Parametric analyses were further performed to clarify the influence of bond width on stress transfer, stiffness evolution, and interfacial performance. The findings provide experimental evidence and numerical reference for understanding the CFRP–masonry interfacial behavior and load-transfer characteristics of traditional grey brick masonry bonded with glutinous rice mortar.

## 2. Materials and Basic Mechanical Properties

### 2.1. Material Preparation

In this study, fired clay grey bricks and glutinous rice mortar were adopted to construct masonry specimens, which were intended to represent a traditional brick masonry material system commonly found in Chinese historic masonry buildings. Glutinous rice mortar is a traditional lime-based organic–inorganic mortar that has been widely used in Chinese historic masonry structures [31,32]. Previous studies have shown that traditional Chinese organic–inorganic mortars were commonly prepared by incorporating organic additives, such as glutinous rice paste, into lime-based inorganic binders [31,33]. The glutinous rice mortar used in this study was prepared using loess, lime, sand, and glutinous rice paste, with the mixture proportion selected with reference to traditional masonry practice and previous studies on glutinous rice mortar.

Based on preliminary mix proportion tests and relevant studies on traditional glutinous rice mortar, a mortar mixture was selected to reproduce the relatively low-strength, low-elastic-modulus, and deformation-compatible characteristics of traditional glutinous rice mortar. The mass ratio of loess, lime, and sand was 1:0.375:1, and glutinous rice paste with a mass concentration of 5% was added during mixing. No modern additives or modification materials were incorporated, so as to preserve the basic mechanical characteristics of the traditional material system.

The mortar specimens were cured for 28 days under laboratory conditions, with a temperature of approximately 25 °C and a relative humidity of approximately 80%. The fired clay grey bricks were stored under the same laboratory conditions before testing.

To obtain the material parameters required for numerical analysis, uniaxial compression tests were conducted on glutinous rice mortar and fired clay grey bricks. Cubic mortar specimens with dimensions of 70.7 mm × 70.7 mm × 70.7 mm were prepared, and six specimens were tested after curing under standard conditions until the specified curing age. The compressive tests of grey bricks were conducted according to Sintered Common Bricks (GB/T 5101–2017) [34] and Test Methods for Wall Bricks (GB/T 2542–2012) [35]. A total of six grey bricks were tested. During the tests, force–displacement curves were recorded, and the density, elastic modulus, and compressive strength of each material were subsequently obtained.

### 2.2. Mechanical Properties of Materials

During compression testing, the glutinous rice mortar specimens initially exhibited elastic behavior, followed by crack initiation and gradual propagation before crushing failure. The specimens maintained a certain degree of integrity after failure, indicating relatively ductile failure characteristics. In contrast, the fired clay grey bricks exhibited brittle failure behavior, with vertical cracking and local spalling occurring before the peak load, followed by rapid crack propagation and fragmentation.

**Figure 1:**
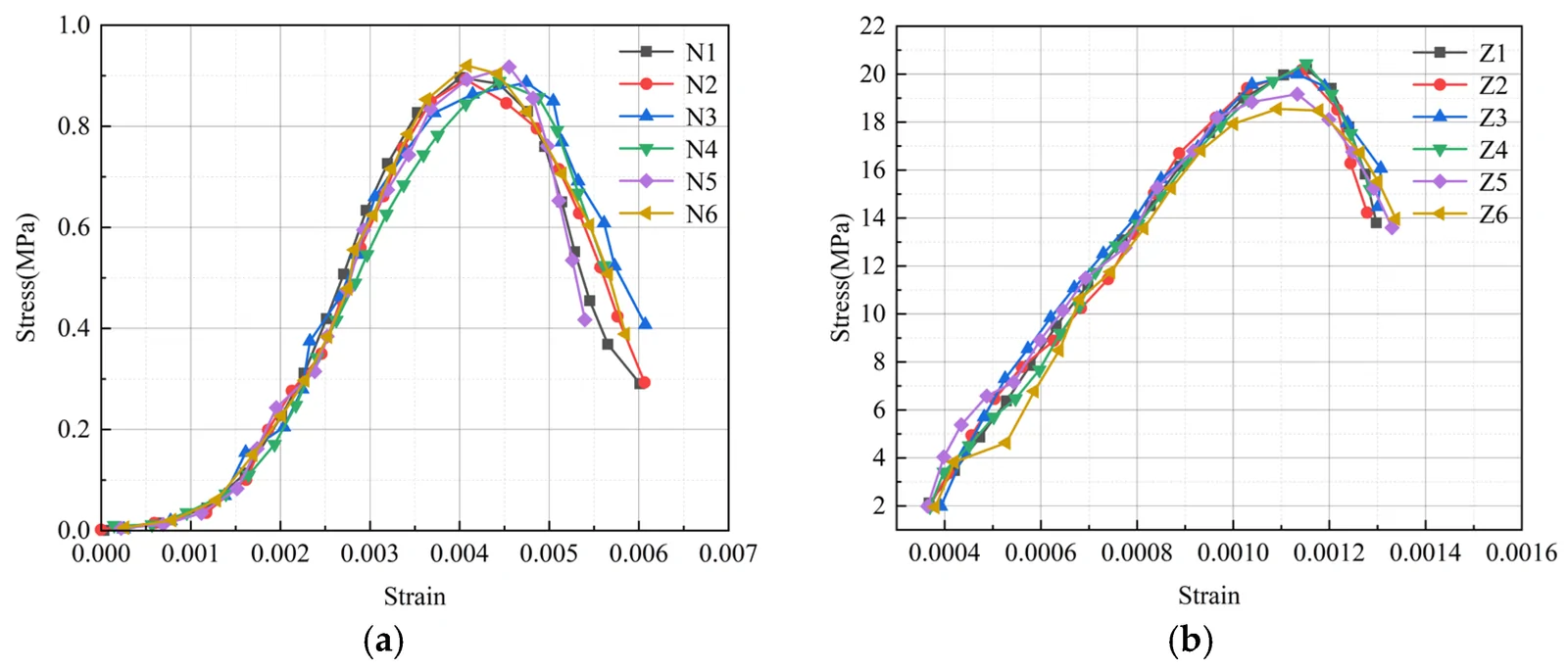
Stress–strain curves of glutinous rice mortar and fired clay grey bricks: (**a**) glutinous rice mortar specimens; (**b**) fired clay grey bricks.

The stress–strain curves of the tested materials are presented in Figure 1, and the corresponding material test results and statistical parameters are summarized in Table 1 and Table 2. The elastic modulus was determined from the slope of the initial linear portion of the stress–strain curves, and the average values obtained from all specimens were adopted as input parameters for subsequent finite element analysis. The average density, compressive strength, and elastic modulus of the glutinous rice mortar were 1750 kg/m^3^, 0.90 MPa, and 165 MPa, respectively. The corresponding values for the fired clay grey bricks were 1713.2 kg/m^3^, 19.8 MPa, and 16 GPa, respectively. These experimentally measured average values were adopted as the material parameters for subsequent finite element analysis because they directly represent the glutinous rice mortar and fired clay grey bricks used in the present specimens, rather than values taken from modern cement-based masonry or literature-based assumptions.

The elastic modulus was used to describe the stiffness and deformation capacity of the brick and mortar components. The fired clay grey bricks exhibited a much higher elastic modulus than the glutinous rice mortar, indicating that the bricks mainly provided stiffness and load-bearing capacity, whereas the mortar joints formed a relatively more deformable phase within the masonry composite. The lower elastic modulus of the glutinous rice mortar implies a greater deformation capacity, which may help accommodate local deformation and improve deformation compatibility between adjacent brick units. Therefore, the elastic response of the masonry specimens should be understood as the combined deformation of the brick units, mortar joints, and brick–mortar interfaces, rather than as the response of a single homogeneous material. This stiffness difference may influence the deformation compatibility of the masonry substrate and the stress-transfer process at the CFRP–masonry interface.

It should be noted that direct tensile and fracture-related properties of the glutinous rice mortar and fired clay grey bricks were not measured in the present study. Therefore, the near-surface masonry damage discussed later was identified mainly from the observed failure modes in the double-shear tests, rather than from independently measured tensile or fracture parameters.

## 3. Experimental Investigation

### 3.1. Specimen Design and Experimental Program

To investigate the interfacial bond behavior between CFRP sheets and glutinous rice mortar-bonded grey brick masonry, double-shear tests were conducted with different bond widths. By symmetrically bonding CFRP sheets on both sides of the masonry specimens and applying axial tensile loading, the force–displacement responses and failure characteristics of the CFRP–masonry interfaces were obtained.

The mechanical behavior of CFRP–masonry interfaces is affected by bond geometry and interface integrity. Therefore, different bond lengths and debonding ratios were considered in the experimental program to evaluate the influence of interface conditions on the interfacial bond behavior. In this study, bond width was selected as one representative geometric parameter controlling the effective load-transfer area. Bond length and debonding ratio were introduced as auxiliary parameters to evaluate whether the observed bond-width-related behavior was affected by different bonding conditions.

**Figure 2:**
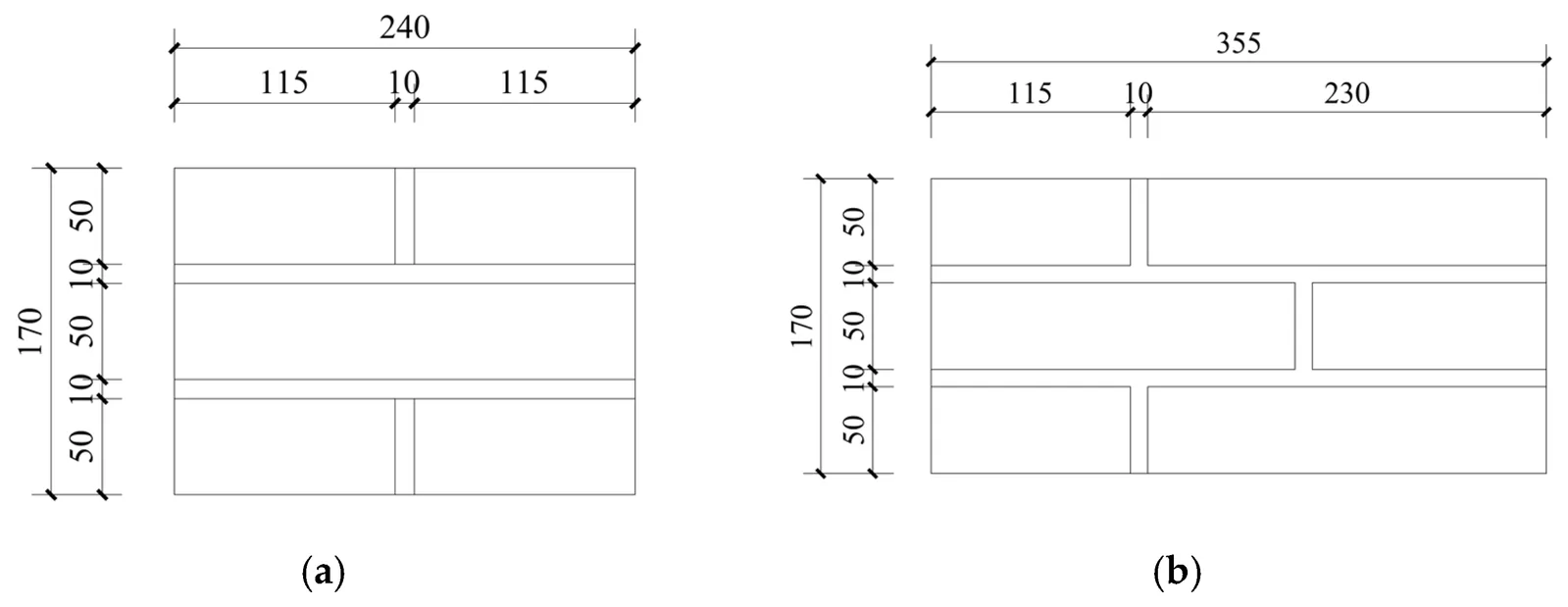

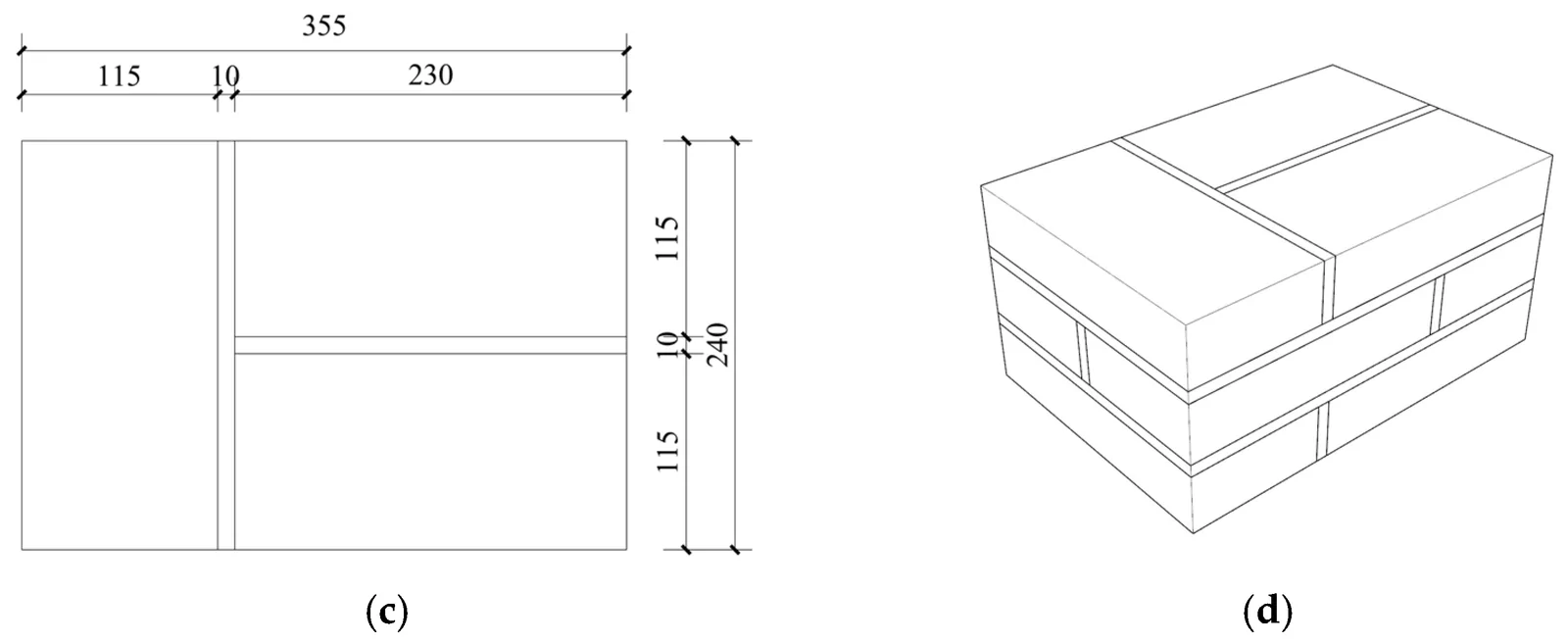
Dimensions and configuration of the double-shear specimens: (**a**) side view; (**b**) front view; (**c**) top view; (**d**) schematic illustration. All dimensions are in mm.

The specimens were constructed using fired clay grey bricks and glutinous rice mortar. Each grey brick had dimensions of 230 mm × 115 mm × 50 mm, and each masonry specimen consisted of nine bricks with a mortar joint thickness of 10 mm, as shown in Figure 2. The assembled masonry specimens were cured under laboratory conditions for 28 days before surface preparation and CFRP bonding. CFRP sheets were bonded to the masonry surface using modified epoxy adhesive. The sheets were symmetrically arranged on both sides of the specimen to form a double-shear loading configuration. To prevent splitting failure of the masonry blocks during loading, U-shaped CFRP wrapping was applied at the sheet ends to ensure effective load transfer within the test region.

**Figure 3:**
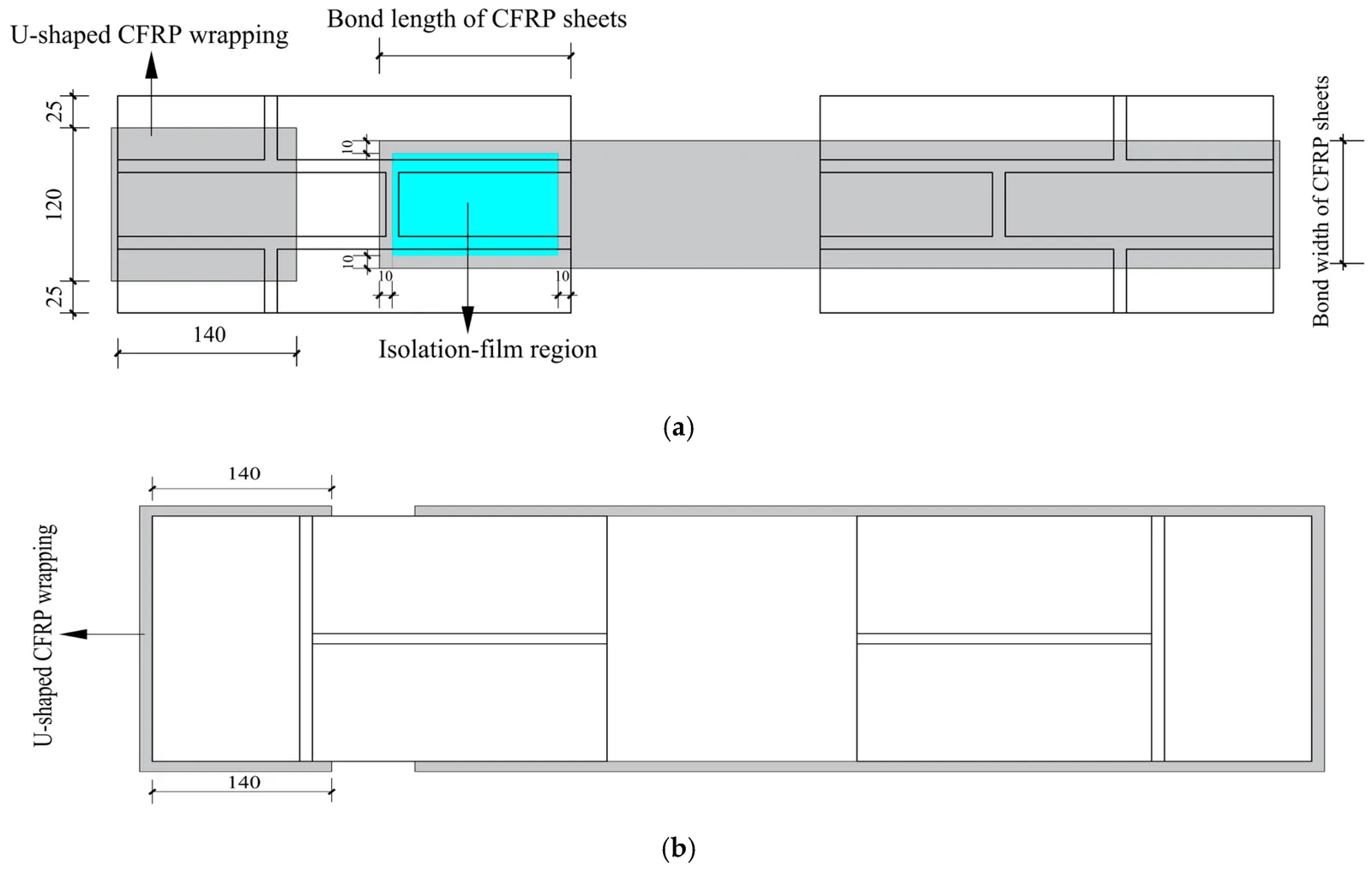
Layout of carbon fiber-reinforced polymer (CFRP) strengthening, U-shaped CFRP wrapping, and isolation-film region: (**a**) side view; (**b**) top view. The blue region indicates the location where the isolation films were uniformly arranged. All dimensions are in mm.

The detailed layout of the CFRP strengthening system, including the bond length, bond width, U-shaped CFRP wrapping, and isolation-film region, is shown in Figure 3. The U-shaped CFRP wrapping was positioned at the sheet ends, with a wrapping length of 140 mm. The isolation films were arranged within the CFRP–masonry bonding region to generate artificial interface defects for specimens with predefined debonding ratios.

The details of the CFRP strengthening system were further specified to ensure the reproducibility of the tests. A single layer of CFRP sheet was used in all strengthened specimens, and the main mechanical properties of the CFRP sheet and modified epoxy adhesive are summarized in Table 3.

Before CFRP bonding, the masonry surface was mechanically polished using an angle grinder to remove surface irregularities. Residual mortar was removed with a scraper, and surface dust was thoroughly cleaned using a clean towel. The CFRP sheets were cut along the principal fiber direction according to the designed bond dimensions.

A two-component modified epoxy adhesive was used for bonding. Component A, the resin matrix, and component B, the curing agent, were mixed at a volume ratio of A:B = 2:1 according to the manufacturer’s instructions. Because the bonding operation was conducted in winter, component A was indirectly heated in a water bath at 60–70 °C before mixing to reduce its viscosity and improve workability, while preventing water from entering the adhesive. The CFRP bonding operation was completed within the workable time of the adhesive.

During bonding, the modified epoxy adhesive was applied continuously and uniformly to the masonry surface. The CFRP sheets were then bonded and repeatedly rolled along the fiber direction using a dedicated roller to remove entrapped air, promote adhesive impregnation of the fiber bundles, and ensure a continuous bonded interface. After CFRP bonding, the strengthened specimens were cured under laboratory conditions for 7 days to ensure sufficient hardening of the adhesive before testing.

A total of 27 test cases were designed, considering three parameters: bond length (L), bond width (B), and debonding ratio ($\eta_{d}$). The bond lengths were set as 100, 150, and 200 mm, while the bond widths were 50, 75, and 100 mm. The debonding ratios were selected as 0%, 10%, and 20%. Two identical specimens were prepared for each parameter combination. The average values of the repeated specimens were used for the comparison of peak-force-related parameters, while representative force–displacement curves were selected to illustrate the typical interfacial response.

The complete specimen matrix, including the individual peak force, peak displacement, observed failure mode, mean value, and coefficient of variation, is summarized in Table 4.

The coefficients of variation in the repeated specimens were generally within a limited range, with several displacement-related values slightly exceeding 5%, which can be attributed to the inherent heterogeneity of traditional masonry materials and the sensitivity of interfacial slip measurements.

Considering that traditional brick masonry buildings may experience deterioration processes such as weathering and salt crystallization during long-term service, local unbonded regions may develop between CFRP sheets and masonry substrates. Therefore, different debonding ratios were introduced to simulate different levels of interface discontinuity. These cases were used to examine the influence of interface conditions on the interfacial bond behavior and to evaluate whether the bond-width-related performance variation was affected by local unbonded regions, rather than being treated as independent research variables.

**Figure 4:**
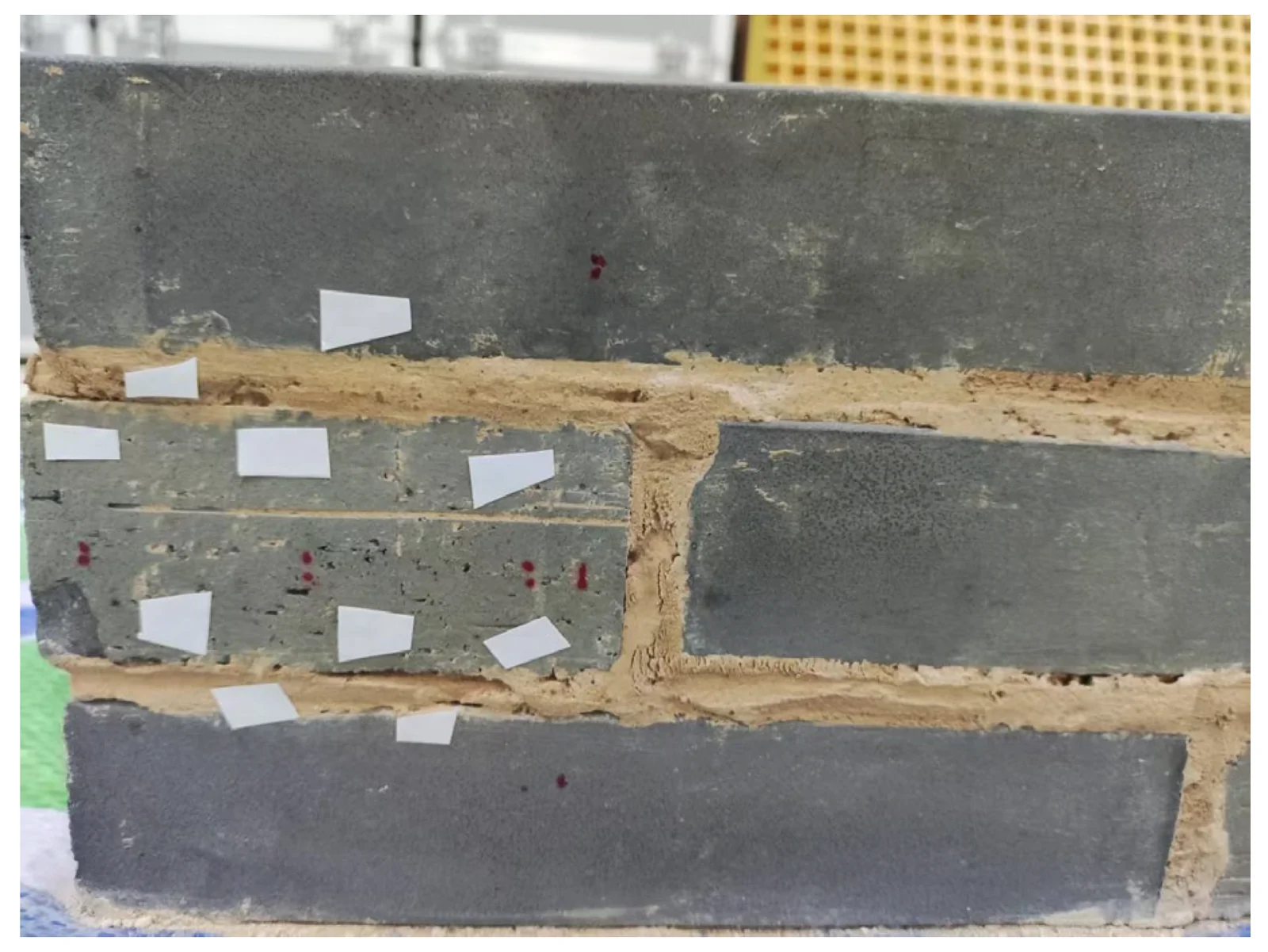
Photographs of artificial interface defects generated by isolation films before CFRP bonding.

The unbonded regions were controlled by placing isolation films at predetermined locations, resulting in specimens with $\eta_{d}$ = 10% and 20%, as shown in Figure 4. The debonding ratio was defined as the ratio of the unbonded area to the designed bonded area. The location of the isolation-film region relative to the loaded end is indicated by the blue region in Figure 3a. The isolation films were arranged on the side CFRP–masonry bonding interface planes near the inner loaded ends, where interfacial shear transfer was expected to develop first during loading. Within the blue region, the isolation films were uniformly arranged to obtain a controlled and repeatable artificial defect distribution. This arrangement was adopted to represent a simplified condition of local interface discontinuities that may occur in practice, where defects may be spatially distributed rather than concentrated at a single point. Therefore, the debonding ratio should be interpreted together with the isolation-film arrangement shown in Figure 3, since the adopted defect location and distribution can influence the interfacial stress-transfer process.

To characterize the strain response of the CFRP sheets during interfacial loading, strain gauges (Jincheng Testing Instrument Factory, Liyang, China) were installed along the fiber direction of the CFRP sheets on both sides of the masonry specimens. The measurement points were arranged along the bonded length, with distances of 10, 50, 90, 140, and 170 mm from the loaded end. The CFRP strain data were continuously recorded during the double-shear tests and were used to evaluate the interfacial load-transfer behavior.

### 3.2. Loading Procedure and Measurement Method

**Figure 5:**
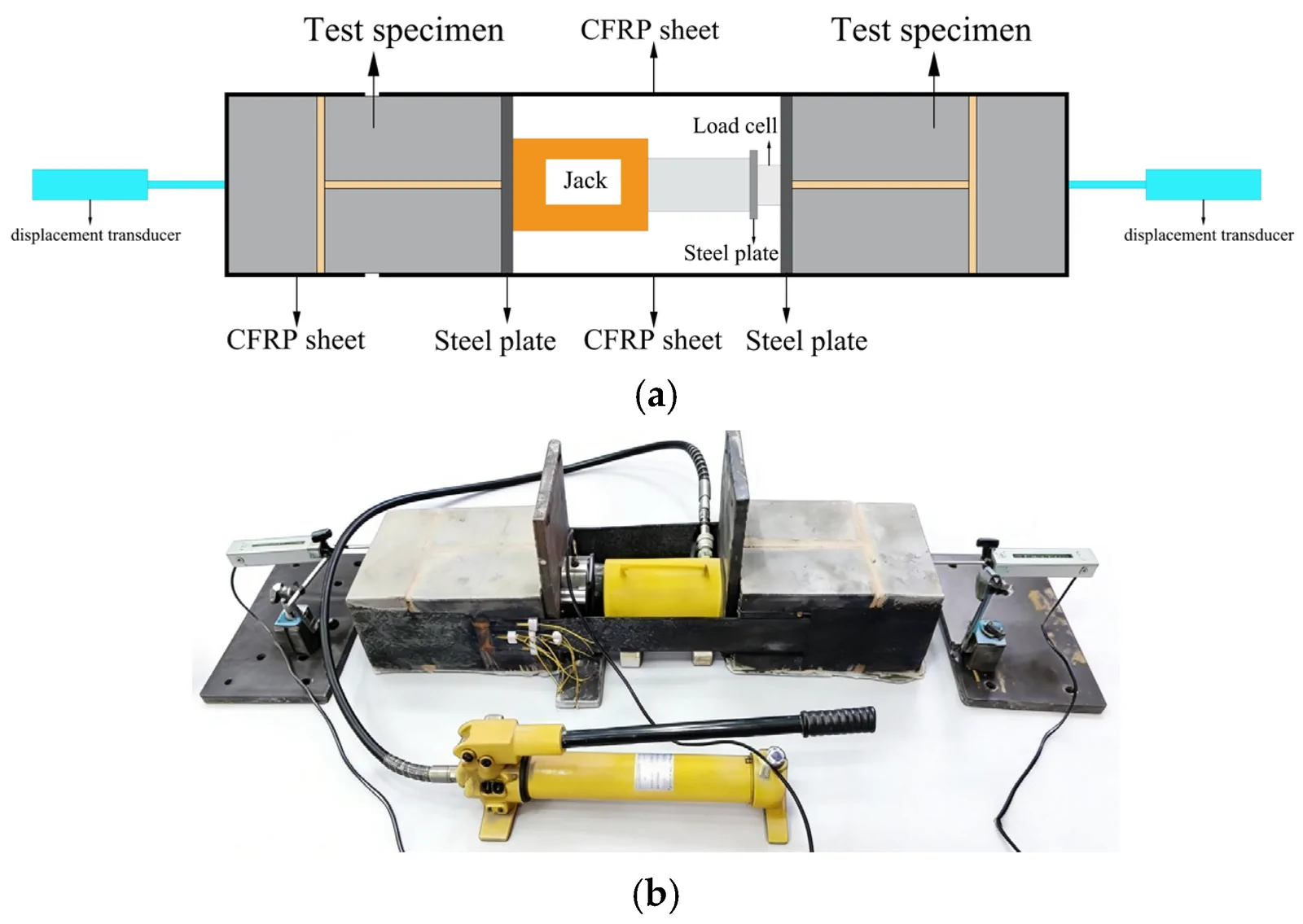
Loading setup for the double-shear test: (**a**) schematic illustration of the double-shear setup; (**b**) photograph of the experimental setup.

**Figure 6:**
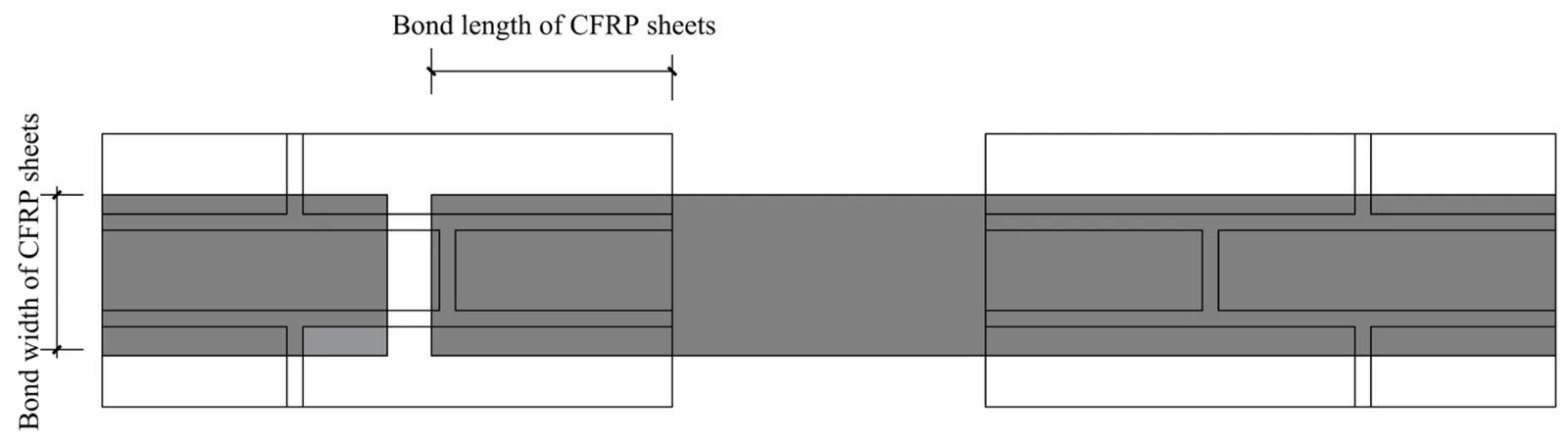
Schematic configuration of CFRP–masonry bonded specimens.

The loading setup for the double-shear test is shown in Figure 5. During the test, a horizontal tensile force was applied to the central loading device, inducing synchronous tensile actions in the CFRP sheets bonded on both sides of the specimen. Consequently, shear transfer was generated along the CFRP–masonry interfaces. Displacement-controlled loading was adopted, and the applied force, displacement, and surface strain of the CFRP sheets were simultaneously recorded throughout the loading process. The CFRP sheets were symmetrically bonded on both sides of the masonry specimens. The bonded length and width were defined according to the dimensions of the CFRP sheets, as illustrated in Figure 6.

In the double-shear configuration, two CFRP–masonry bonded interfaces were activated simultaneously. The force recorded by the load cell was the total actuator force applied to the specimen. Since the present study focused on the shear response of a single bonded interface, the force reported in the force–displacement curves and in Table 4 was taken as one half of the total actuator force and is denoted as P.

The displacement reported in the force–displacement curves was measured by displacement transducers installed on both sides of the masonry specimen. During loading, the masonry specimen moved relative to the CFRP sheets; therefore, the measured displacement represents the relative displacement between the CFRP sheet and the masonry substrate, rather than the cross-head movement of the testing machine. This displacement includes the initial tightening deformation of the CFRP sheet and the subsequent interfacial slip between the CFRP sheet and the masonry substrate.

To characterize the interfacial stress transfer behavior, strain gauges were arranged along the bonded length of the CFRP sheets to obtain the strain distributions at different loading stages. Meanwhile, displacement measurement devices were installed on both sides of the specimens to record the relative deformation between the CFRP sheets and the masonry substrate during loading.

The force–displacement responses of the interfaces were obtained using the load acquisition and displacement measurement systems. Combined with the CFRP strain distributions, the interfacial bond behavior and load-transfer characteristics were evaluated.

### 3.3. Failure Modes and Observations

**Figure 7:**
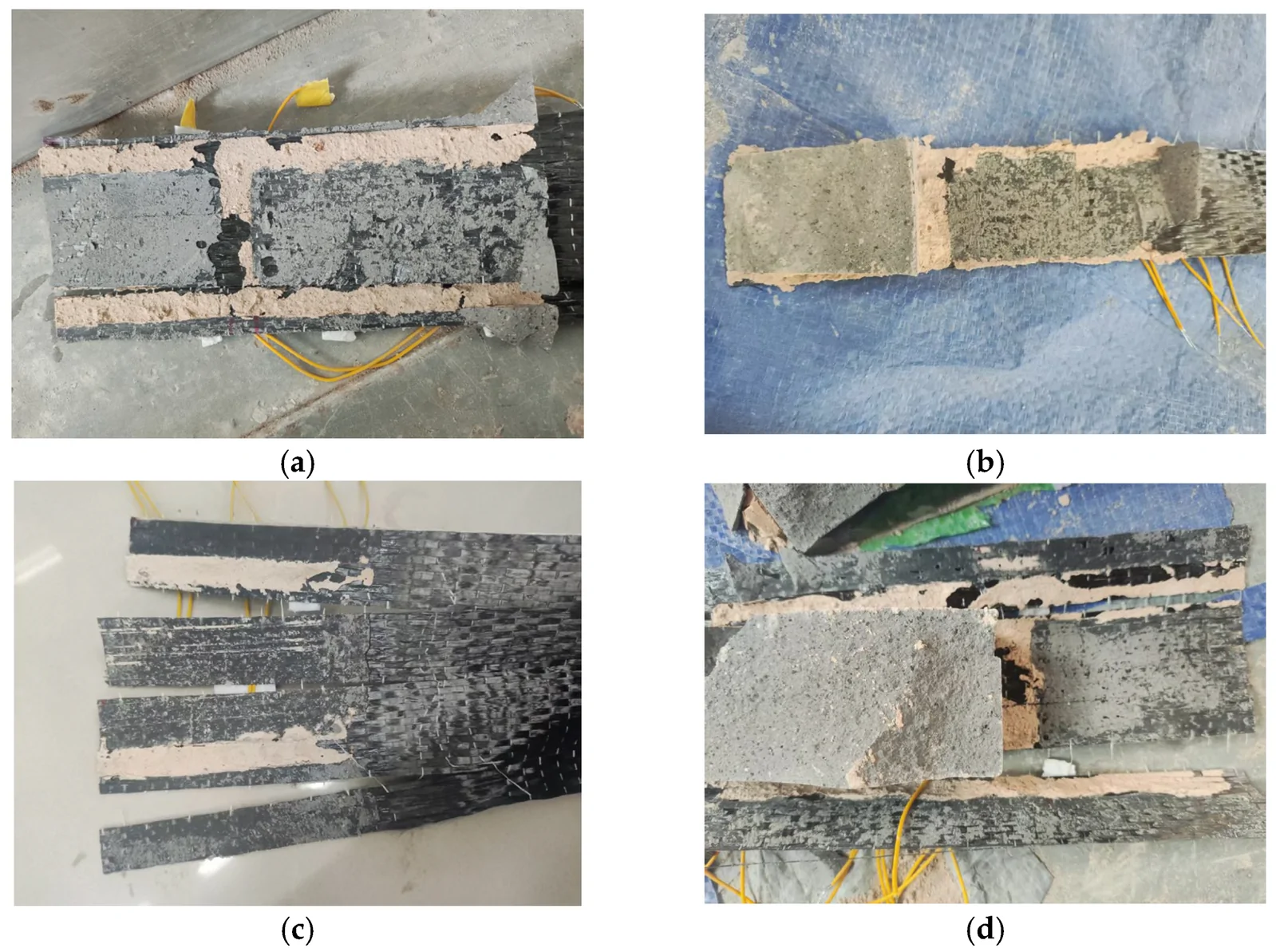
Failure modes of CFRP-strengthened traditional masonry interfaces: (**a**) interfacial debonding failure; (**b**) coupled failure of CFRP debonding and near-surface masonry damage; (**c**) CFRP sheet rupture failure; (**d**) combined masonry damage and interfacial debonding failure.

During the double-shear tests, the CFRP–glutinous rice mortar-bonded grey brick masonry interfaces exhibited different failure characteristics under increasing load. At the initial loading stage, the CFRP sheets and masonry substrate remained well bonded and deformed compatibly, resulting in an approximately linear increase in the force–displacement response. With further loading, local debonding initiated near the loaded end, accompanied by localized fiber rupture in some specimens. After reaching the peak force, interface separation and near-surface masonry damage developed, resulting in several typical failure modes, including interfacial debonding, coupled interfacial debonding and near-surface masonry damage, CFRP sheet rupture, and combined masonry damage and interfacial debonding, as shown in Figure 7.

Figure 7a presents a typical interfacial debonding failure, where separation occurred between the CFRP sheet and masonry substrate, accompanied by detachment of part of the surface mortar. Figure 7b shows a coupled failure mode involving CFRP debonding and near-surface masonry damage, where local separation of the CFRP sheet occurred together with shear cracking in the surface masonry layer. Figure 7c corresponds to CFRP sheet rupture, where the CFRP fibers fractured along the loading direction while some bonded regions remained attached to the masonry surface. Figure 7d illustrates the combined failure mode of masonry damage and interfacial debonding, where interface separation and masonry cracking occurred simultaneously during loading.

Overall, the observed failure modes were mainly characterized by interfacial debonding accompanied by local damage in the near-surface masonry layer. These results indicate that the load-carrying performance of CFRP-strengthened traditional masonry interfaces was primarily controlled by the interfacial bond behavior. Moreover, similar failure characteristics were observed under different bond widths, bond lengths, and debonding ratios, providing a basis for further evaluating the influence of bond width on interfacial load-carrying performance.

### 3.4. Interfacial Load-Carrying Behavior Under Different Bond Width Conditions

The nominal bond stress was calculated from the mean peak force of the two repeated specimens for each configuration and added to Table 4 as a normalized index. As shown in Table 4, the mean peak force generally increased with increasing bond width, whereas the nominal bond stress did not show a consistent increasing trend. For example, when L = 100 mm and ηd = 0%, increasing the bond width from 50 mm to 100 mm increased the mean peak force from 4.15 kN to 7.47 kN, while the nominal bond stress decreased from 0.83 MPa to 0.75 MPa. A similar trend was observed for specimens with L = 150 mm and ηd = 0%, where the nominal bond stress decreased from 0.83 MPa to 0.76 MPa.

These results indicate that the increase in peak force was mainly associated with the enlarged bonded area. Therefore, in this study, the bond width is interpreted primarily as a geometric parameter affecting the load-carrying capacity of the CFRP–masonry interface. Bond length and debonding ratio were introduced to provide different bonded-geometry and interface-integrity conditions for examining the consistency of the bond-width-related trend. In addition, increasing the debonding ratio generally reduced the nominal bond stress, indicating that artificial interface defects weakened the effective stress transfer across the interface.

**Figure 8:**
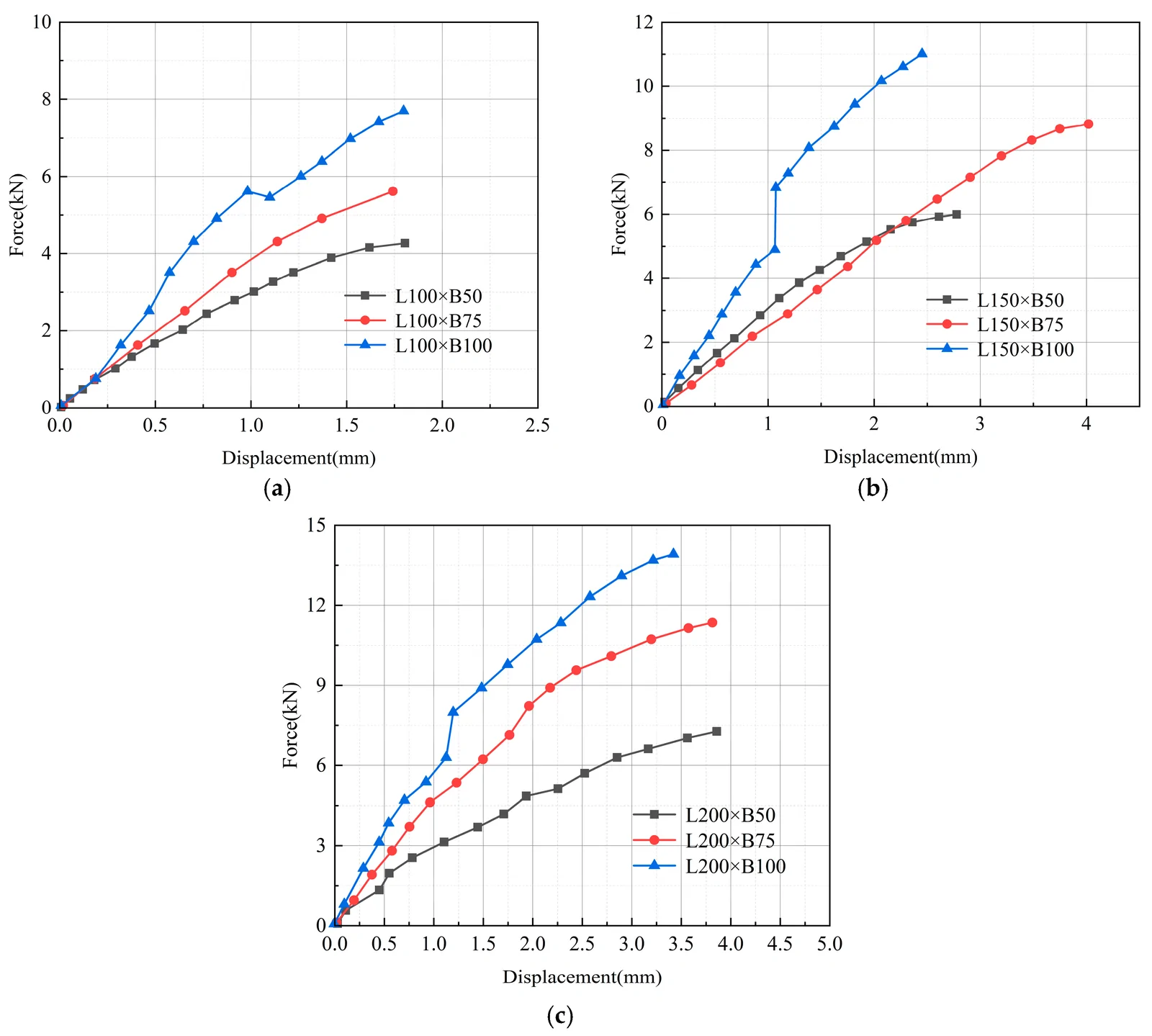
Representative force–displacement responses of CFRP-strengthened traditional masonry interfaces with different bond widths: (**a**) L = 100 mm, B = 50–100 mm; (**b**) L = 150 mm, B = 50–100 mm; (**c**) L = 200 mm, B = 50–100 mm.

**Figure 9:**
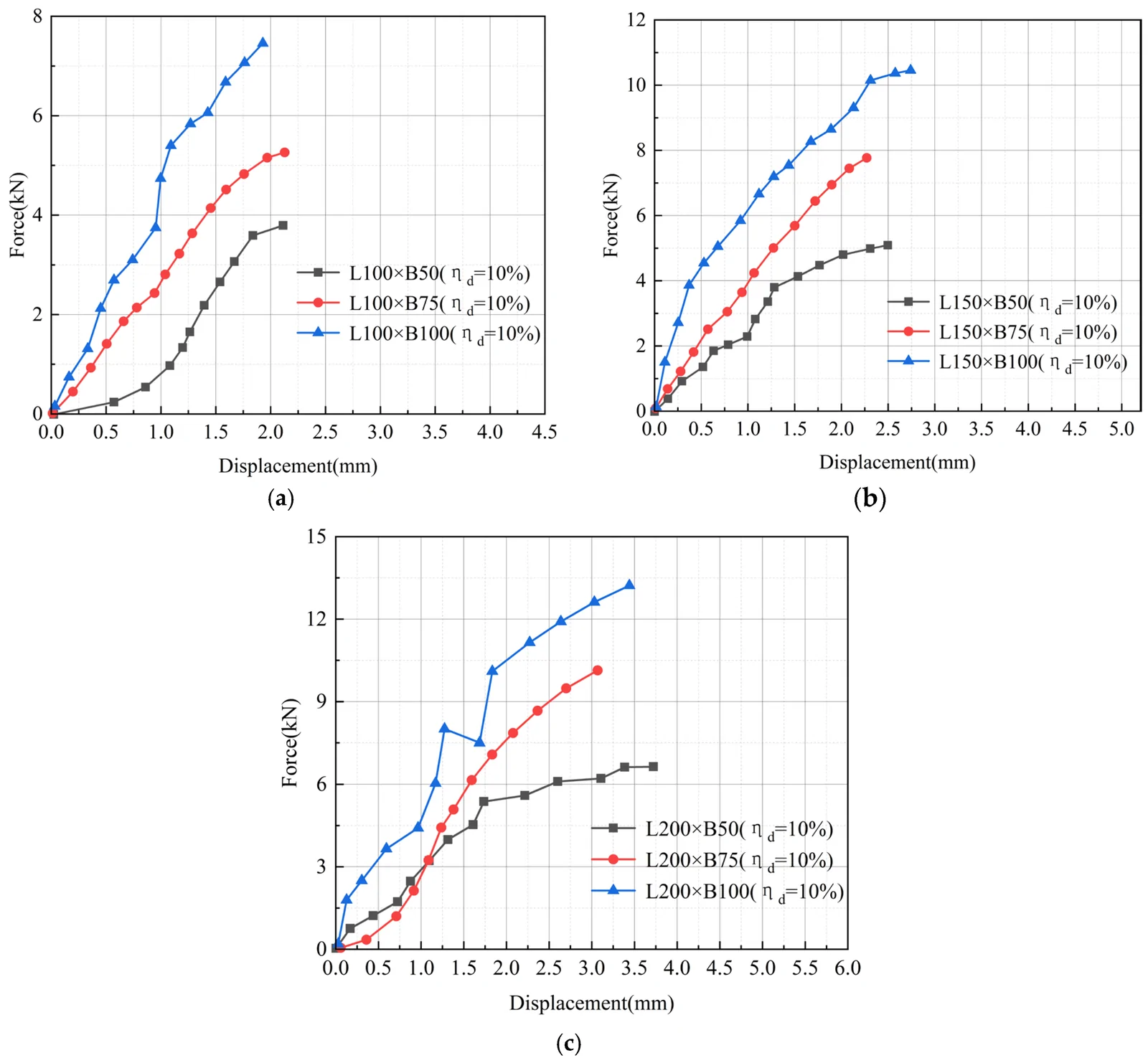
Representative force–displacement responses of CFRP-strengthened traditional masonry interfaces with a 10% debonding ratio: (**a**) L = 100 mm, B = 50–100 mm; (**b**) L = 150 mm, B = 50–100 mm; (**c**) L = 200 mm, B = 50–100 mm.

**Figure 10:**
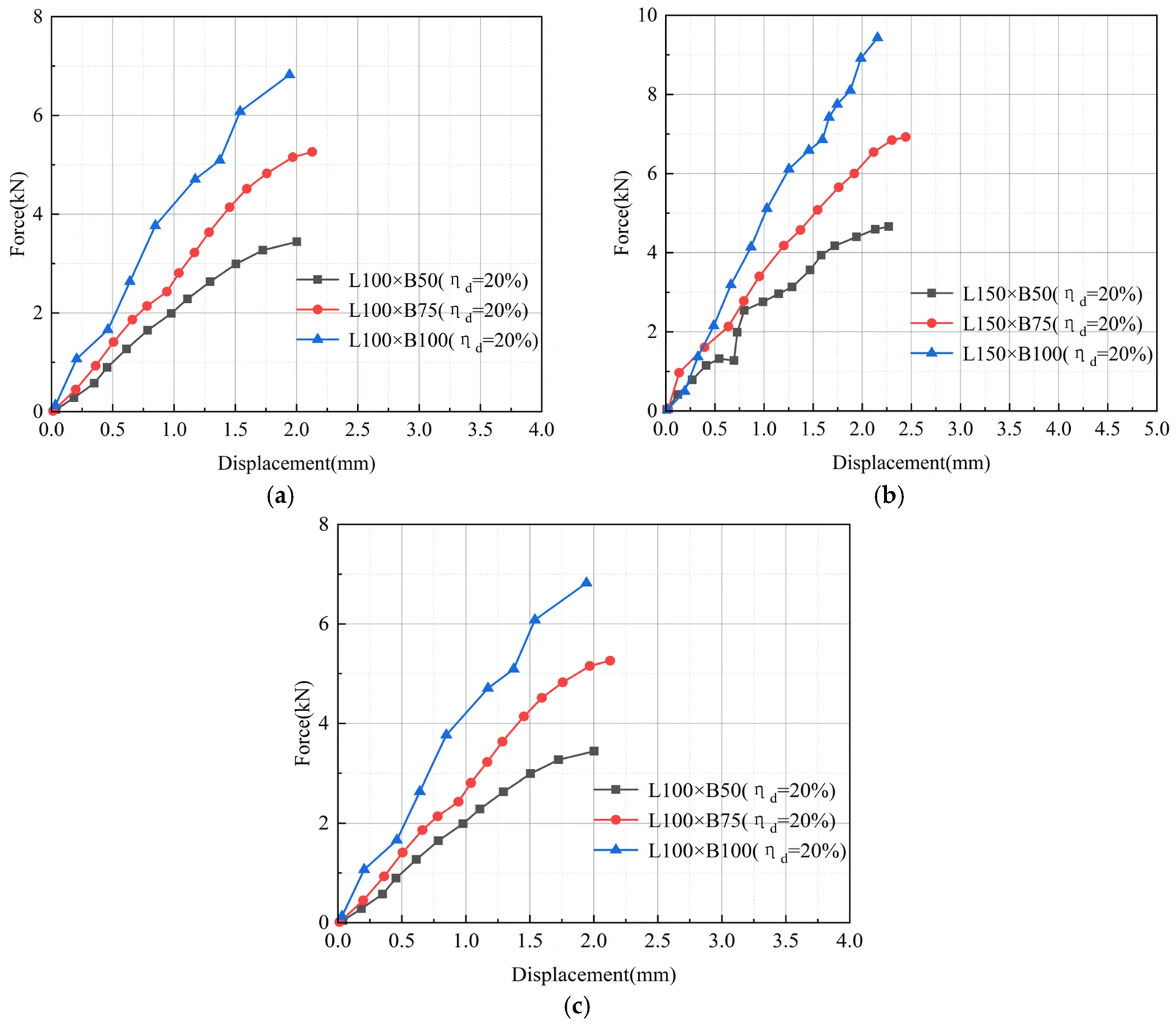
Representative force–displacement responses of CFRP-strengthened traditional masonry interfaces with a 20% debonding ratio: (**a**) L = 100 mm, B = 50–100 mm; (**b**) L = 150 mm, B = 50–100 mm; (**c**) L = 200 mm, B = 50–100 mm.

Figure 8, Figure 9 and Figure 10 show representative force–displacement responses selected from the two repeated specimens for each configuration. The representative curve was selected from the specimen with a complete and stable testing record and a failure mode consistent with the main failure characteristics of that configuration. These curves were used to illustrate the typical interfacial response process under different bond widths, bond lengths, and interface integrity conditions. The experimental results demonstrate that increasing the CFRP bond width consistently improves the interfacial load-carrying capacity, and similar variation trends were observed under different bond lengths and interface integrity conditions. Although bond length and debonding ratio also affect the absolute load-carrying capacity of the interface, they were mainly considered herein to provide different bonding conditions for examining the observed bond-width-related response.

Taking the specimens with a bond length of L = 100 mm as an example, when the bond width increased from 50 mm to 100 mm, the peak shear forces per bonded interface of specimens with intact interfaces, 10% debonding ratio, and 20% debonding ratio increased by 80.3%, 96.1%, and 98.4%, respectively. Similar enhancement trends were observed for specimens with bond lengths of 150 mm and 200 mm, with capacity increases ranging from 83.6% to 114.8%. These results indicate that increasing the bond width effectively improves the interfacial load-carrying capacity and that the width-related trend remains consistent within the investigated parameter range.

Further analysis of the force–displacement curves show that increasing the bond width shifts the curves upward and increases the initial slope, indicating an increase in the initial interfacial stiffness. Under the same displacement level, specimens with larger bond widths sustained higher forces, demonstrating improved load-transfer efficiency due to the enlarged effective bonded area. Although debonding regions reduced the overall force level and introduced local fluctuations, the capacity ranking among different bond widths remained unchanged, indicating that bond width has an important influence in the interfacial load-carrying behavior within the investigated range.

### 3.5. CFRP Strain Response During Interfacial Loading

**Figure 11:**
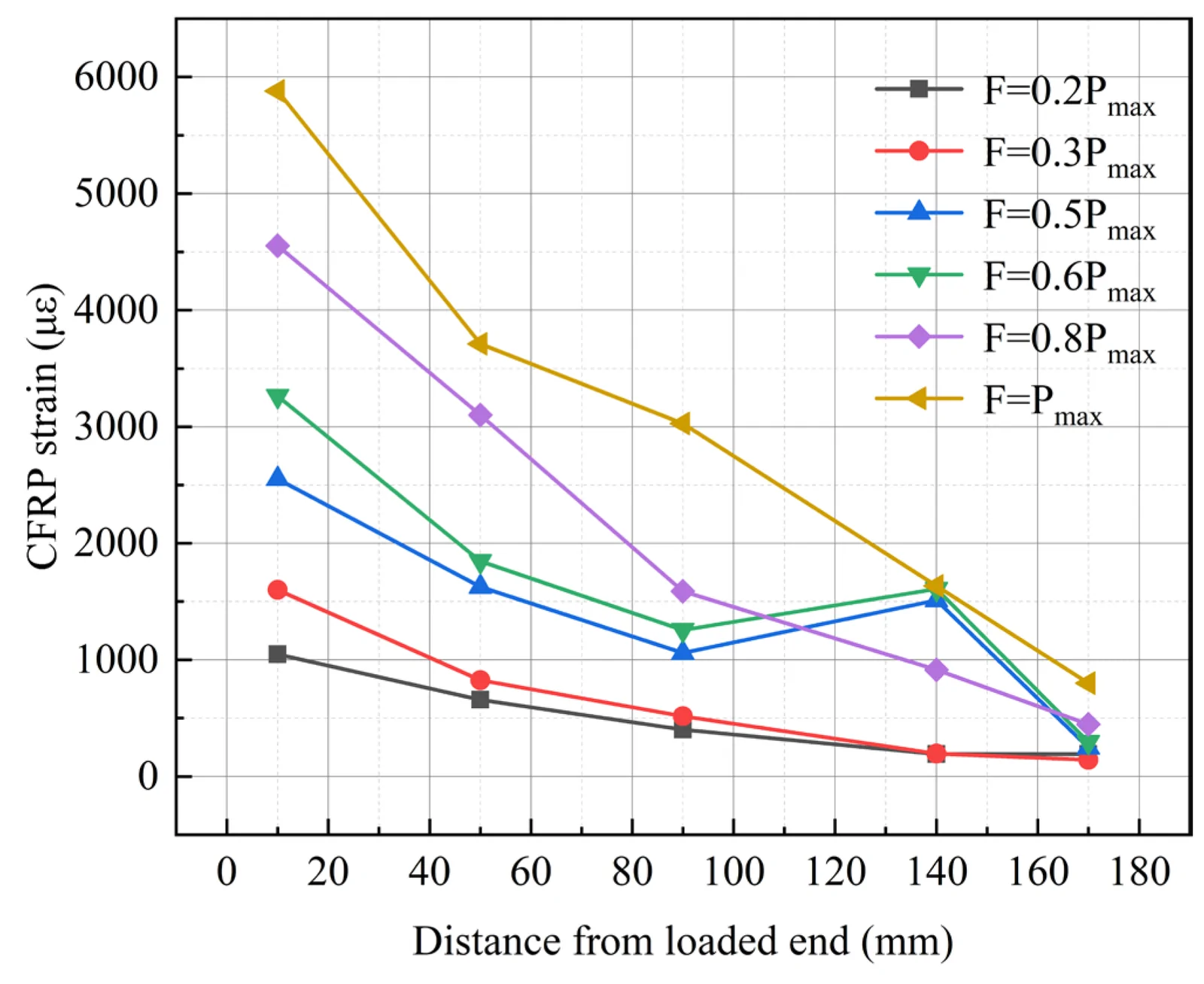
CFRP strain distribution at different loading levels.

To further investigate the mechanical response of the CFRP–glutinous rice mortar masonry interface, strain gauges were installed along the fiber direction of the CFRP sheet to monitor the deformation response during loading. Considering that the specimen with a larger bonded area exhibited more complete load-transfer characteristics, a representative specimen with a bond length of 200 mm and a bond width of 100 mm under an intact interface condition (L200 × B100, ηd = 0%) was selected for analysis. Figure 11 presents the CFRP strain distributions at different loading levels, corresponding to 20%, 30%, 50%, 60%, 80%, and 100% of the peak interfacial capacity.

As shown in Figure 11, the CFRP strain exhibited a gradual decrease from the loaded end toward the free end throughout the loading process. During the initial loading stage, the CFRP sheet and masonry substrate maintained compatible deformation, and the strain level within the bonded region increased progressively with increasing load while the overall distribution pattern remained stable. With further loading, the CFRP strain near the loaded end continued to increase, whereas the strain growth in regions farther from the loaded end was relatively limited. This indicates that the interfacial shear transfer gradually developed from the loaded end toward the bonded region, forming a continuous load-transfer process between the CFRP sheet and masonry substrate.

When the applied load approached the peak interfacial capacity, obvious strain concentration occurred near the loaded end, where the strain growth rate became higher than that in other regions. This phenomenon was consistent with the interfacial debonding and near-surface masonry damage observed during the tests, indicating that the local interface region experienced relatively higher shear transfer demand near the peak load stage.

Based on the measured CFRP strain profiles, the average local interfacial shear stress between adjacent strain gauges was further estimated from the strain gradient. For two adjacent strain gauges located at $x_{i}$ and $x_{i+1}$, the average local interfacial shear stress can be expressed as

(1) $$\tau (x)=\frac{E_{f}t_{f}d\varepsilon_{f}}{dx}$$

(2) $$\tau (x_{i})=E_{f}t_{f}\frac{\varepsilon_{i+1}-\varepsilon_{i}}{x_{i+1}-x_{i}}$$

where $E_{f}$ and $t_{f}\;$ are the elastic modulus and nominal thickness of the CFRP sheet, respectively; $\varepsilon_{i}\;$ and $\varepsilon_{i+1}$ are the CFRP strains measured at two adjacent positions. The strain values were converted from microstrain to dimensionless strain before calculation. Therefore, the calculated $\tau \left(x_{i}\right)$ represents the average interfacial shear stress over the interval between two adjacent strain gauges.

As shown in Table 5, the estimated average local interfacial shear stress generally increased with the applied load level. At the peak load, the highest value was obtained in the 10–50 mm interval, indicating that the region close to the loaded end carried a relatively high shear-transfer demand. This is consistent with the CFRP strain distribution shown in Figure 11, where higher strain and a larger strain gradient were observed near the loaded end.

The strain profiles also indicate that the main load-transfer region was concentrated near the loaded end at the early loading stage and gradually extended toward the free end as the applied load increased. Since the identification of a numerical transfer length is sensitive to the selected threshold criterion, the present analysis focuses on the qualitative evolution of the load-transfer region rather than a fixed transfer-length value. In addition, local fluctuations were observed in some intervals, indicating that the strain-gradient-based shear stress values should be interpreted as interval-averaged estimates rather than continuous local shear-stress distributions.

Additional strain profiles from specimens with different bond widths and repeated tests were also examined. These profiles showed similar overall characteristics, namely that the CFRP strain was relatively higher near the loaded end and gradually decreased toward the free end. Therefore, only the representative L200 × B100 specimen is presented in Figure 11 to illustrate the typical strain-development and load-transfer process, while avoiding excessive repetition of similar strain-profile results. The strain comparison is used as supporting evidence for the representative load-transfer pattern rather than as a complete quantitative comparison among all bond-width conditions.

## 4. Numerical Simulation and Parametric Analysis

### 4.1. Finite Element Model Development

The double-shear test results demonstrated that the failure of most specimens was dominated by interfacial debonding between the CFRP sheet and masonry substrate, sometimes accompanied by local damage in the near-surface masonry layer. Therefore, the finite element analysis focused on reproducing the interfacial bond behavior and load-transfer characteristics of the CFRP–masonry interface.

**Figure 12:**
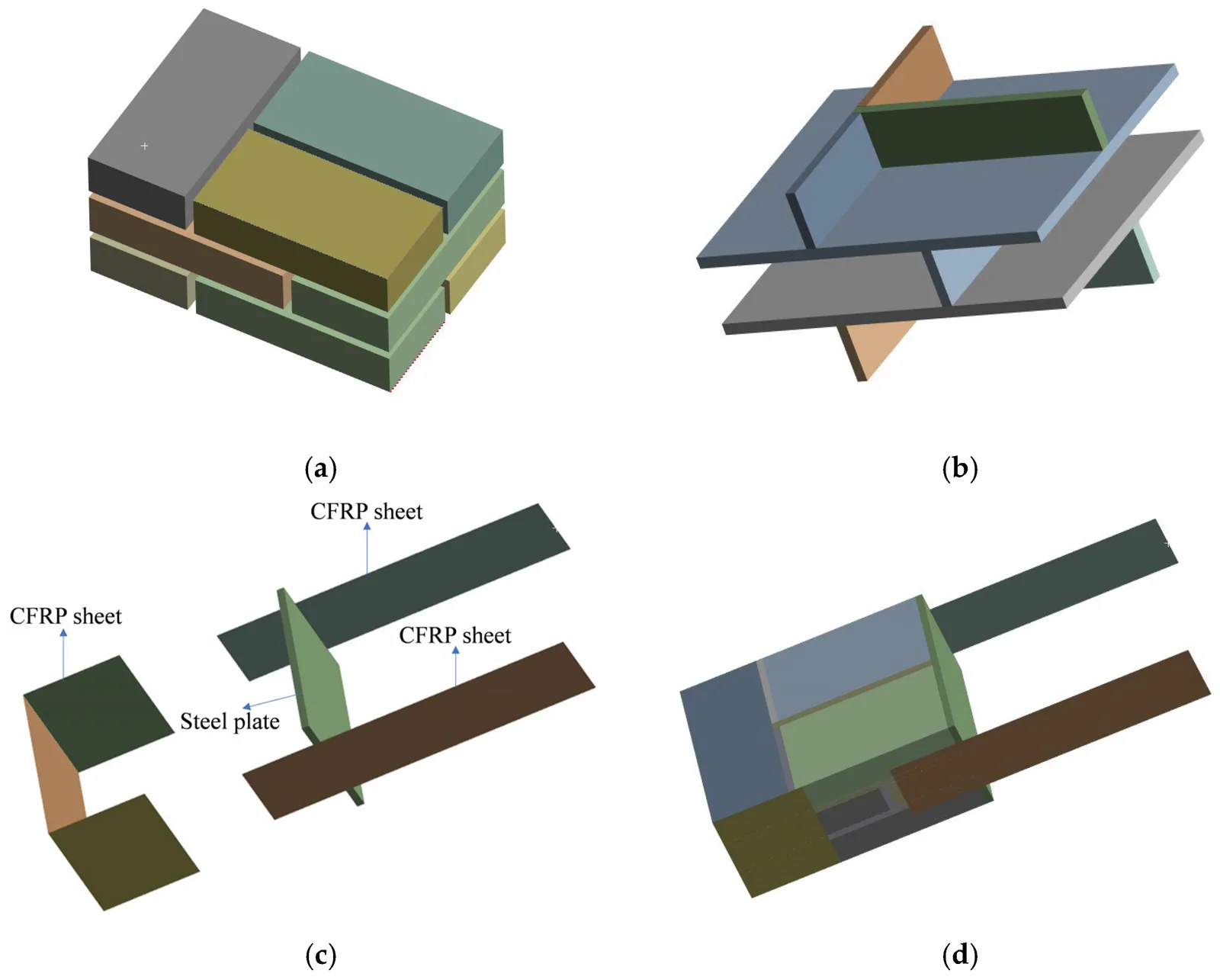
Finite element models of CFRP-strengthened traditional masonry: (**a**) fired clay grey brick model; (**b**) glutinous rice mortar model; (**c**) CFRP sheet and loading steel plate models; (**d**) overall model.

**Figure 13:**
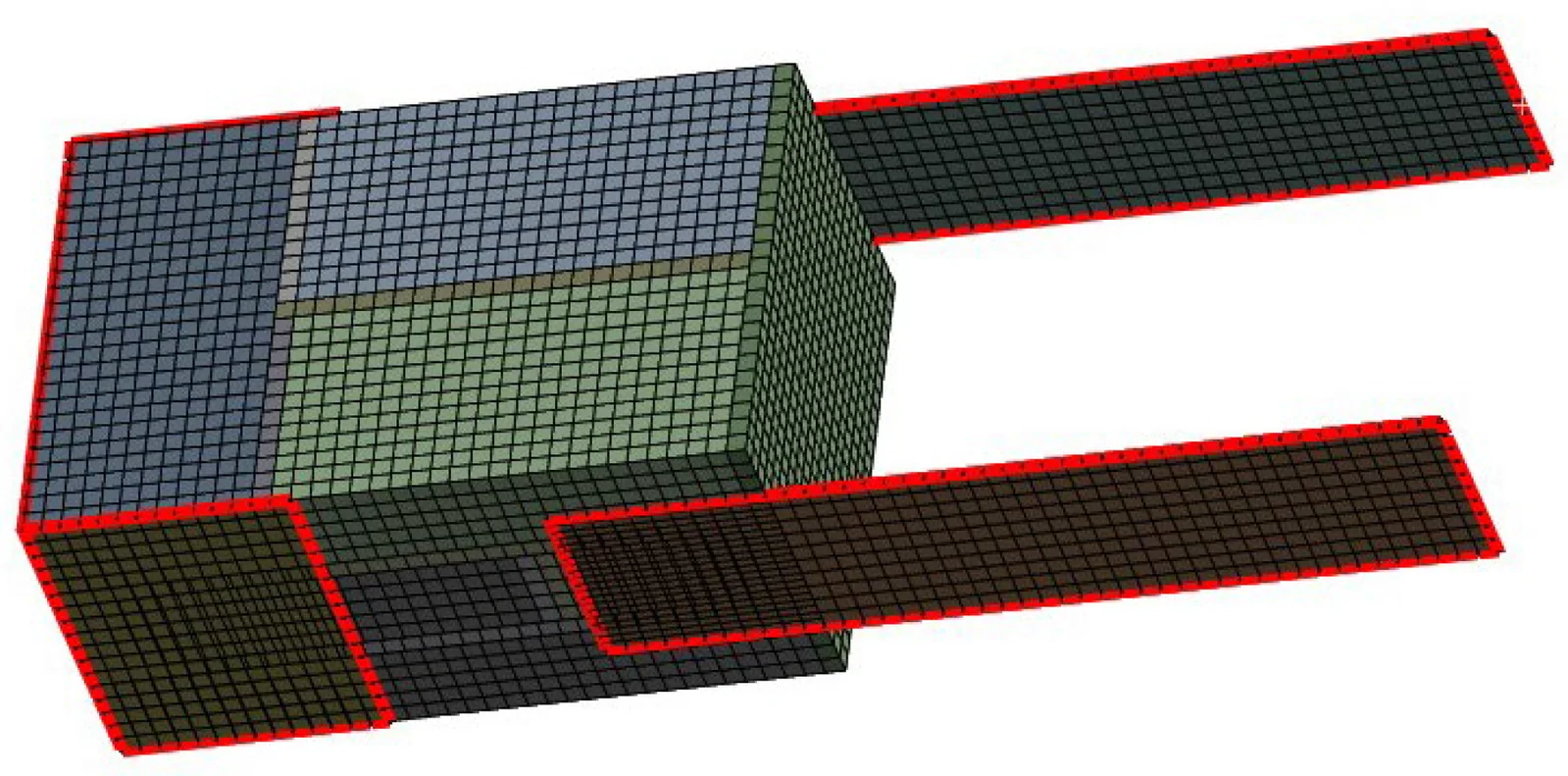
Overall finite element model.

A finite element model consistent with the double-shear test configuration was developed to further investigate the interfacial behavior between CFRP sheets and glutinous rice mortar-bonded grey brick masonry. A separated modeling approach was adopted, in which the fired clay grey bricks, glutinous rice mortar, CFRP sheets, and loading steel plates were modeled individually, as shown in Figure 12 and Figure 13. The dimensions of the grey bricks were 230 mm × 115 mm × 50 mm, the mortar joint thickness was 10 mm, and the CFRP sheet thickness was 0.111 mm. Solid elements were used to simulate the grey bricks and glutinous rice mortar, while shell elements were adopted for the CFRP sheets. The interface between the CFRP sheet and masonry substrate was simulated using zero-thickness interface elements to represent the interfacial bonding behavior and debonding process. A swept meshing method was used, and a typical element size of approximately 10 mm was adopted in the bonded region and adjacent masonry substrate. An independent mesh-sensitivity analysis was not conducted in the present study. The adopted mesh size was selected to provide a refined discretization in the bonded region and adjacent masonry substrate, and the adequacy of the mesh was evaluated through the comparison between the numerical and experimental force–displacement responses in Section 4.2.

**Figure 14:**
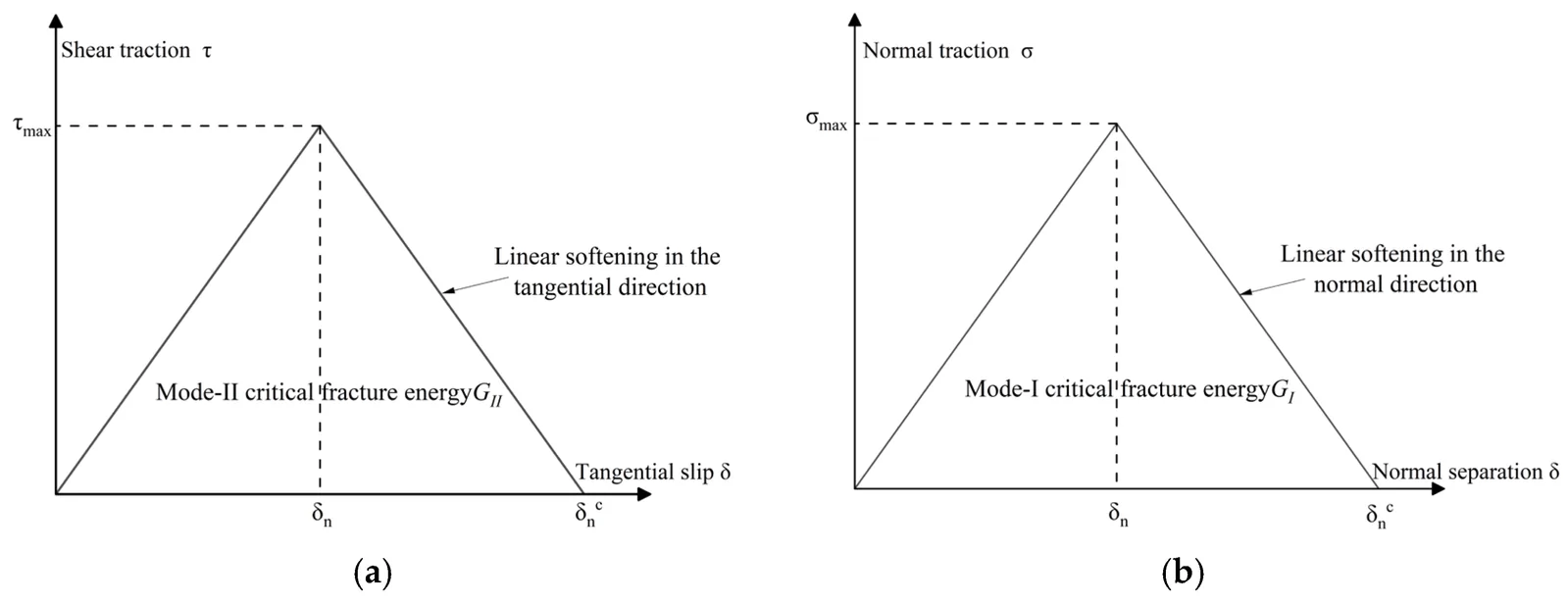
Bilinear traction–separation relationship of the CFRP–masonry interface: (**a**) tangential behavior; (**b**) normal behavior.

Accurate simulation of interfacial bonding and debonding behavior is essential for reproducing the mechanical response of CFRP-strengthened masonry interfaces. In this study, an interface formulation available in the finite element software ANSYS 19.0 (ANSYS Inc., Canonsburg, PA, USA) was adopted to describe the traction–separation relationship between the CFRP sheet and masonry substrate, enabling simulation of interfacial bonding and debonding behavior under loading [36,37,38]. A bilinear traction–separation relationship was employed, as shown in Figure 14. The main parameters included the maximum normal contact stress, maximum equivalent tangential contact stress, normal fracture energy, tangential fracture energy, and artificial damping coefficient. The cohesive parameters were treated as calibrated interface parameters rather than directly measured local bond–slip properties.

The interface parameters were determined based on the results of the reference experimental specimens. The maximum equivalent tangential contact stress was calculated according to the peak force:

(3) $$\tau_{max}=\frac{P_{max}}{A},$$

where $P_{max}$ is the peak shear force transferred by one bonded interface (kN), obtained as one half of the peak total actuator force recorded by the load cell; A is the effective bonded area of one CFRP–masonry interface (mm^2^); and $\tau_{max}$ is the maximum equivalent tangential contact stress (MPa). In the calculation, $P_{max}$ was converted from kN to N.

The value calculated using Equation (3) represents an average bonded-area stress. Therefore, it was used as an equivalent tangential cohesive strength in the simplified cohesive-zone model. This equivalent treatment was adopted to reproduce the global force–displacement response and the overall interfacial stress-transfer characteristics, rather than to determine a local bond–slip law.

The equivalent cohesive parameters were determined from the experimental response of the representative L100 × B50 specimen used for numerical calibration, where L and B denote the bond length and bond width, respectively, and were then kept unchanged in the subsequent numerical simulations. The maximum equivalent tangential contact stress was calculated using Equation (3), while the tangential fracture energy was determined from the force–displacement response during the complete interfacial debonding stage. Considering that the double-shear tests were mainly governed by shear transfer, the normal interface parameters were determined according to the ratio between shear and tensile interface properties reported in previous FRP interface studies [39]. The adopted interface parameters are summarized in Table 6.

The material parameters used in the numerical model were taken from the experimental measurements reported in the preceding sections. The fired clay grey bricks and glutinous rice mortar were described using ideal elastoplastic constitutive models, while the CFRP sheets were assumed to behave linearly elastically before interface failure. The fired clay grey bricks and glutinous rice mortar joints were modelled as separate solid components according to the specimen geometry. No separate cohesive law was assigned to the brick–mortar joints; the cohesive-zone model was used for the CFRP–masonry bonding interface.

The boundary conditions were simplified based on the experimental loading method. The edges of the CFRP sheets were fixed, and displacement-controlled loading was applied to the surface of the steel plate. The displacement increment was 0.2 mm for each loading step, and a total of 40 loading steps were used, corresponding to a total imposed displacement of 8 mm.

For the specimens with artificial interface defects, the deboned regions were simulated by partitioning the CFRP shell elements according to the designed defect area. The CFRP shell elements in the deboned regions were retained, whereas the contact relationship between these CFRP shell elements and the masonry substrate was suppressed. Therefore, the deboned regions were represented as initial unbonded interface defects without contact or cohesive stress transfer.

It should be noted that the present finite element model was developed mainly to reproduce the global force–displacement response, CFRP strain distribution, and dominant CFRP–masonry interfacial debonding behaviour. The near-surface masonry damage observed in some tests was not explicitly simulated as discrete cracking or fracture propagation of the brick or mortar substrate. Instead, the influence of local substrate damage was reflected in an equivalent manner through the degradation and failure of the cohesive interface. Therefore, the numerical results should be interpreted as simulations of the dominant interfacial debonding response and stress-transfer characteristics, rather than as a detailed fracture analysis of the masonry substrate.

### 4.2. Experimental–Numerical Comparison

**Figure 15:**
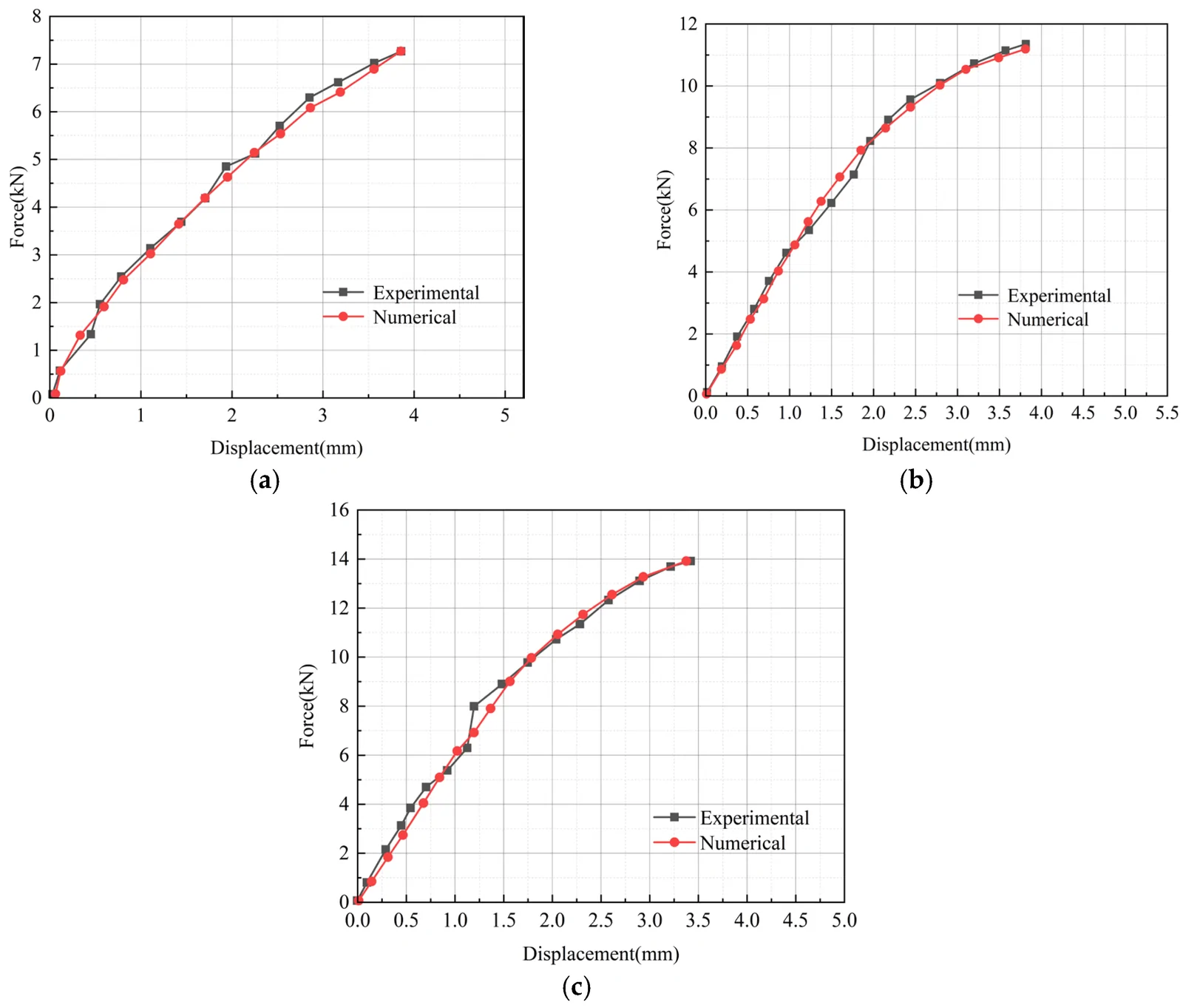
Comparison of experimental and numerical force–displacement responses for representative CFRP–masonry interfaces: (**a**) L200 × B50; (**b**) L200 × B75; (**c**) L200 × B100.

To examine the ability of the developed finite element model to reproduce the main experimental response, the L200 × B50, L200 × B75, and L200 × B100 specimens were selected for experimental–numerical comparison, as shown in Figure 15. These three specimens were not used for determining the cohesive parameters. As described in Section 4.1, the equivalent cohesive parameters were determined from the experimental response of the L100 × B50 specimen and were then kept unchanged in the subsequent numerical simulations. Therefore, the comparison with the L200 specimens was used to evaluate the model’s ability to reproduce the global force–displacement response and overall load-transfer characteristics after the interface parameters had been fixed.

As shown in Figure 15, the numerical results reproduced the overall variation trends of the experimental curves, including the initial linear stage, peak load stage, and post-peak stiffness degradation stage. Under different bond width conditions, the numerical and experimental curves showed good consistency, indicating that the developed finite element model can reasonably describe the load-transfer behavior of the CFRP–masonry interface.

To further quantify the agreement between the experimental and numerical results, the peak force, peak displacement, and initial stiffness of the representative L200 specimens were compared, as summarized in Table 7. The comparison shows that the numerical model reproduced the peak force and peak displacement of the selected specimens with relatively small errors, while the initial stiffness was also captured with reasonable consistency. The slightly larger stiffness errors may be attributed to the simplified equivalent interface model and the inherent heterogeneity of the traditional masonry substrate. These results provide quantitative support for the experimental–numerical comparison shown in Figure 15.

The numerical results further indicated that stress concentration mainly occurred near the loaded end and gradually extended along the bonded region with increasing displacement, which was consistent with the interfacial debonding and near-surface masonry damage observed in the double-shear tests. This agreement demonstrates that the numerical model can effectively capture the interfacial stress transfer characteristics and overall mechanical response of CFRP-strengthened traditional masonry interfaces. After this experimental–numerical comparison, the finite element model was further employed for parametric analysis of bond width effects.

### 4.3. Numerical Investigation of Load-Transfer Characteristics Under Different Bond Width Conditions

The experimental results demonstrated that the influence trend of bond width on the interfacial load-carrying performance remained consistent under different interface conditions. Therefore, the verified finite element model was further employed to extend the bond width range and evaluate the influence of bond width on interfacial capacity, stiffness variation, and load-transfer characteristics.

Based on the verified model, a parametric analysis was conducted to further evaluate the role of bond width. To examine the influence of bond width under a consistent bonding condition, all extended numerical cases were established with a constant bond length of L = 200 mm and an intact interface condition. In addition to the experimentally investigated bond widths of B = 50, 75, and 100 mm, four additional cases with bond widths of B = 62.5, 87.5, 112.5, and 125 mm were considered. The detailed parameters of the extended cases are summarized in Table 8.

**Figure 16:**
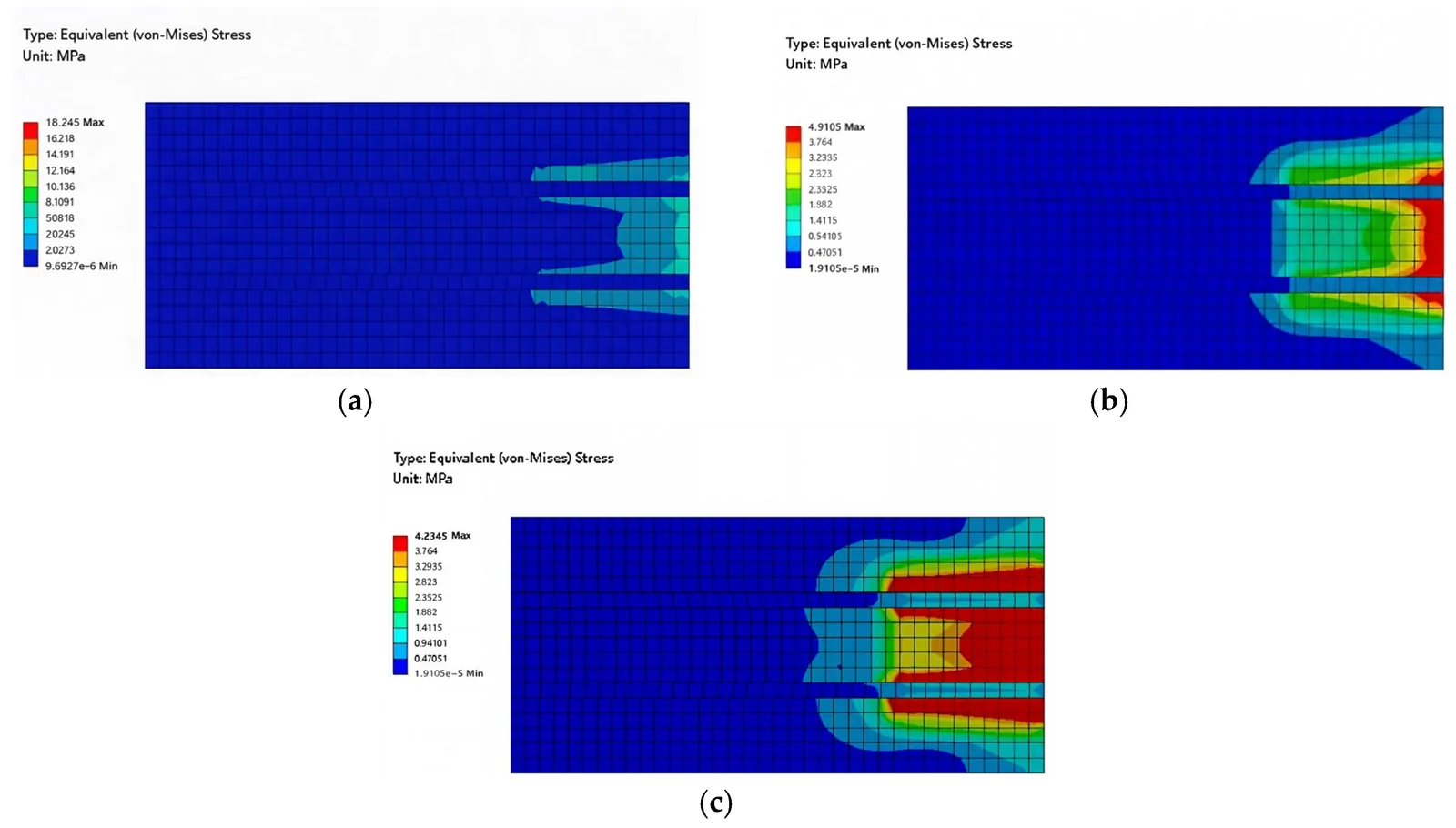
Stress distribution at different loading stages: (**a**) initial elastic stage; (**b**) local slip stage; (**c**) failure stage.

Figure 16 presents the stress distribution at different loading stages, which is used to characterize the load-transfer process and interfacial response of the CFRP–masonry interface. According to the numerical results, the loading process of the CFRP–masonry interface can be divided into three stages: the elastic stage, nonlinear load-transfer stage, and interfacial debonding stage.

During the elastic stage, the CFRP sheet and masonry substrate maintained compatible deformation, and the force–displacement response exhibited an approximately linear trend. The stress distribution within the bonded region was relatively uniform during the initial loading stage. With increasing displacement, nonlinear interfacial response gradually developed near the loaded end, accompanied by local stress redistribution and interfacial slip, resulting in a reduction in the slope of the force–displacement curve. As the peak force was approached, the stress concentration region gradually extended along the bonded length, and interfacial separation occurred, eventually leading to complete debonding. This stress-transfer process was consistent with the debonding behavior observed in the double-shear tests.

**Figure 17:**
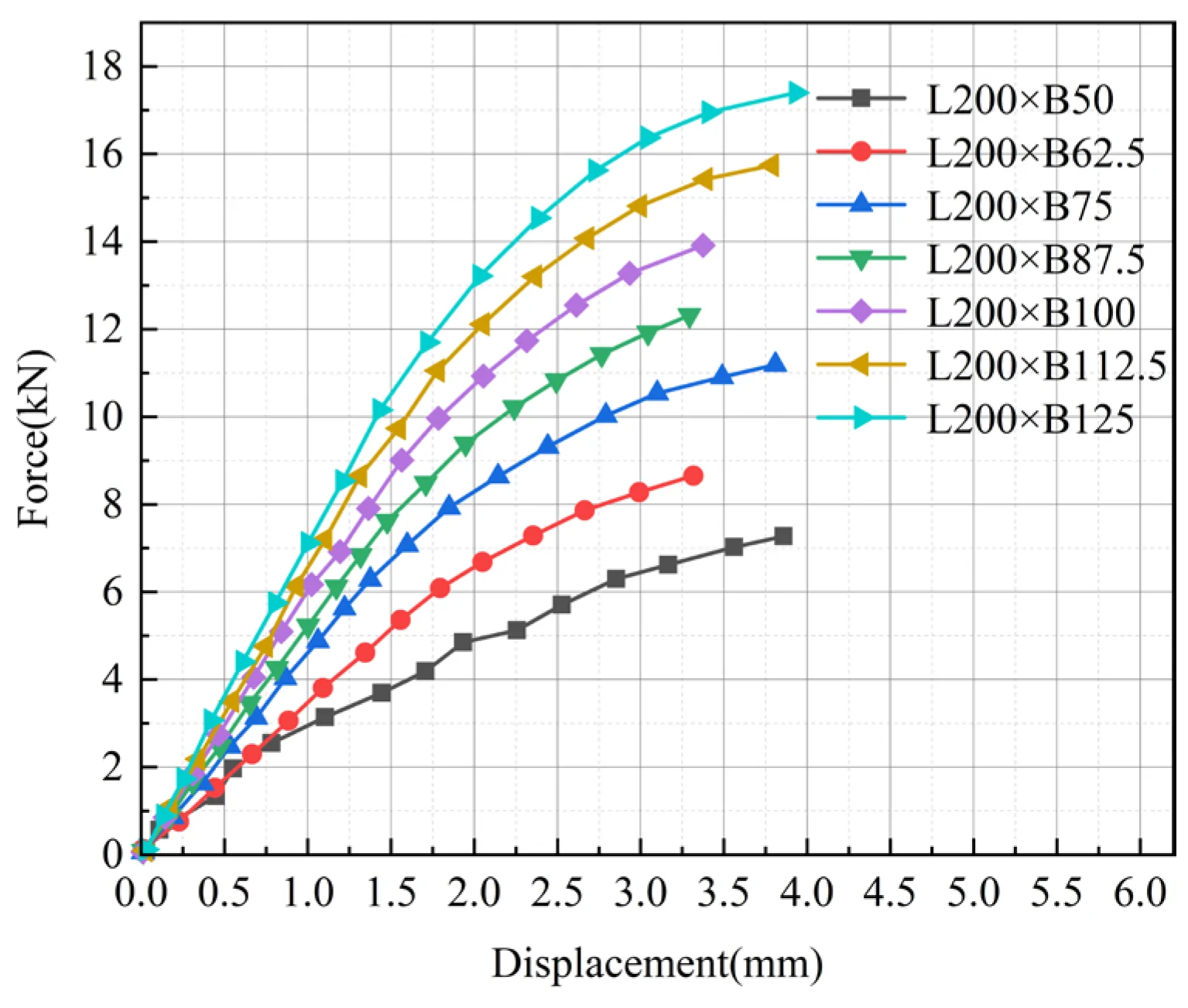
Force–displacement responses of CFRP-strengthened traditional masonry interfaces with different bond widths.

Figure 17 shows the force–displacement responses of the interface with different bond widths. Overall, all cases exhibited similar nonlinear response characteristics, while the curves shifted upward with increasing bond width. Bond width exhibited a significant influence on the ultimate interfacial capacity. For a constant bond length of L = 200 mm, increasing the bond width from 50 mm to 125 mm increased the ultimate capacity from 7.27 kN to 17.40 kN, corresponding to an increase of approximately 139%. The ultimate capacities for bond widths of 62.5, 75, 87.5, 100, 112.5, and 125 mm were approximately 8.65, 11.19, 12.32, 13.92, 15.74, and 17.40 kN, respectively. These results indicate that, within the investigated range, the ultimate interfacial capacity increased approximately linearly with increasing bond width. It should be noted that this nearly linear increase is partly associated with the increase in bonded area under the adopted equivalent cohesive-zone model. Therefore, the width-related increase in numerical capacity should not be interpreted as an independent enhancement of the local interfacial bond property.

From a mechanical perspective, increasing the bond width enlarges the effective load-transfer region between the CFRP sheet and masonry substrate, allowing wider CFRP sheets to participate in load transfer over a larger bonded area. However, this effect should be interpreted as a geometry-related stress-transfer mechanism within the adopted model and tested parameter range, rather than as a direct improvement in the intrinsic local bond strength of the CFRP–masonry interface.

**Figure 18:**
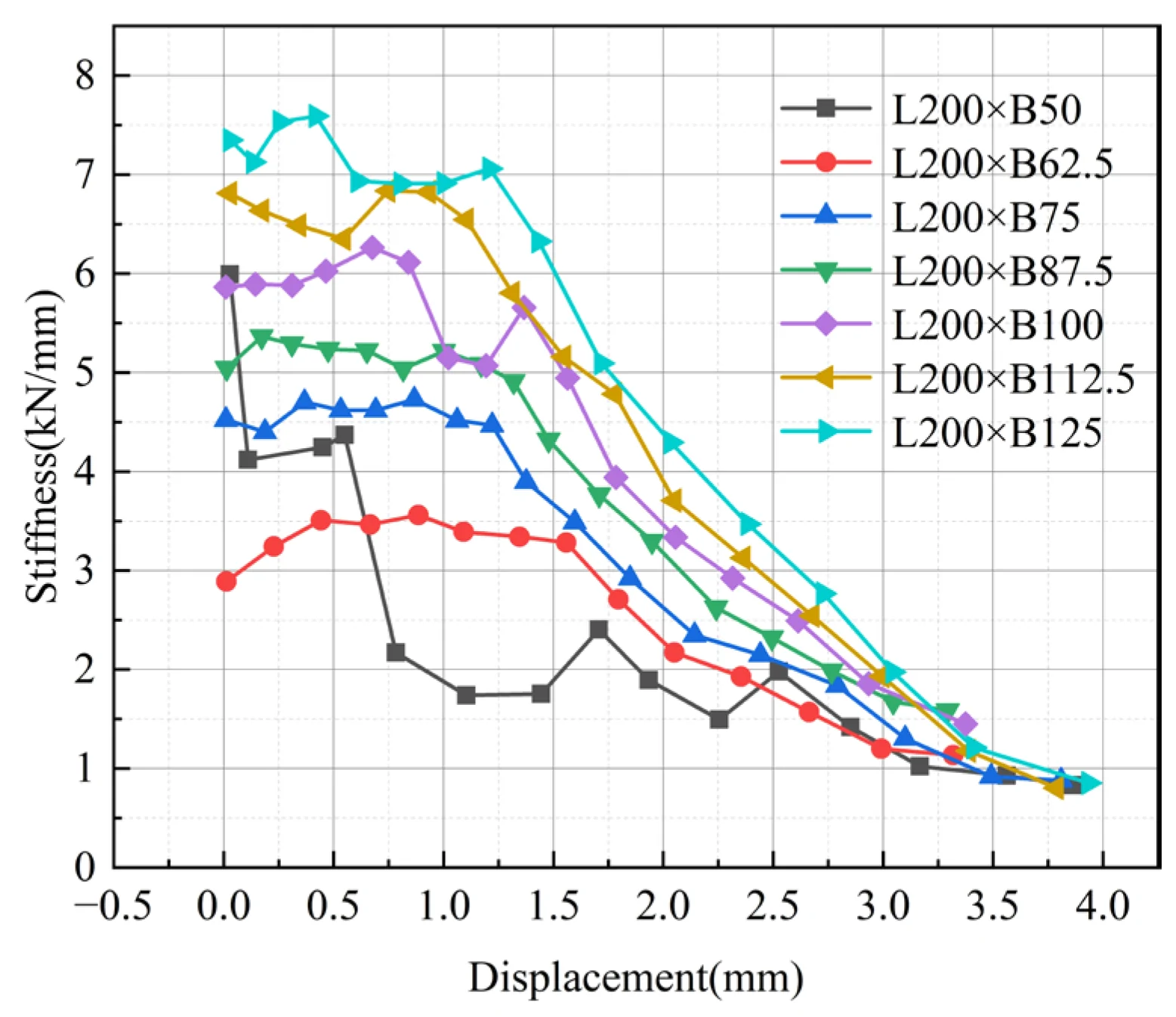
Variation in interfacial stiffness with displacement for different bond widths.

Figure 18 presents the evolution of interfacial stiffness with displacement for different bond widths. The stiffness curves were obtained by calculating the first derivative of the force–displacement relationship and were used to characterize the variation in interfacial stiffness during loading. During the initial loading stage, all cases exhibited relatively high stiffness, and specimens with larger bond widths showed higher initial stiffness, indicating improved early-stage load-transfer capability. With increasing displacement, nonlinear interfacial response gradually developed near the loaded end, resulting in a continuous decrease in interface stiffness and increasing differences among specimens with different bond widths. Compared with narrower CFRP sheets, specimens with larger bond widths maintained higher stiffness over a wider displacement range, demonstrating that increasing bond width contributes to maintaining interfacial stiffness during loading.

At the final stage, the stiffness of all cases gradually decreased to a relatively low level, indicating that the contribution of interfacial bonding to load transfer was reduced under large deformation conditions. At this stage, the influence of bond width on stiffness became less significant.

**Figure 19:**
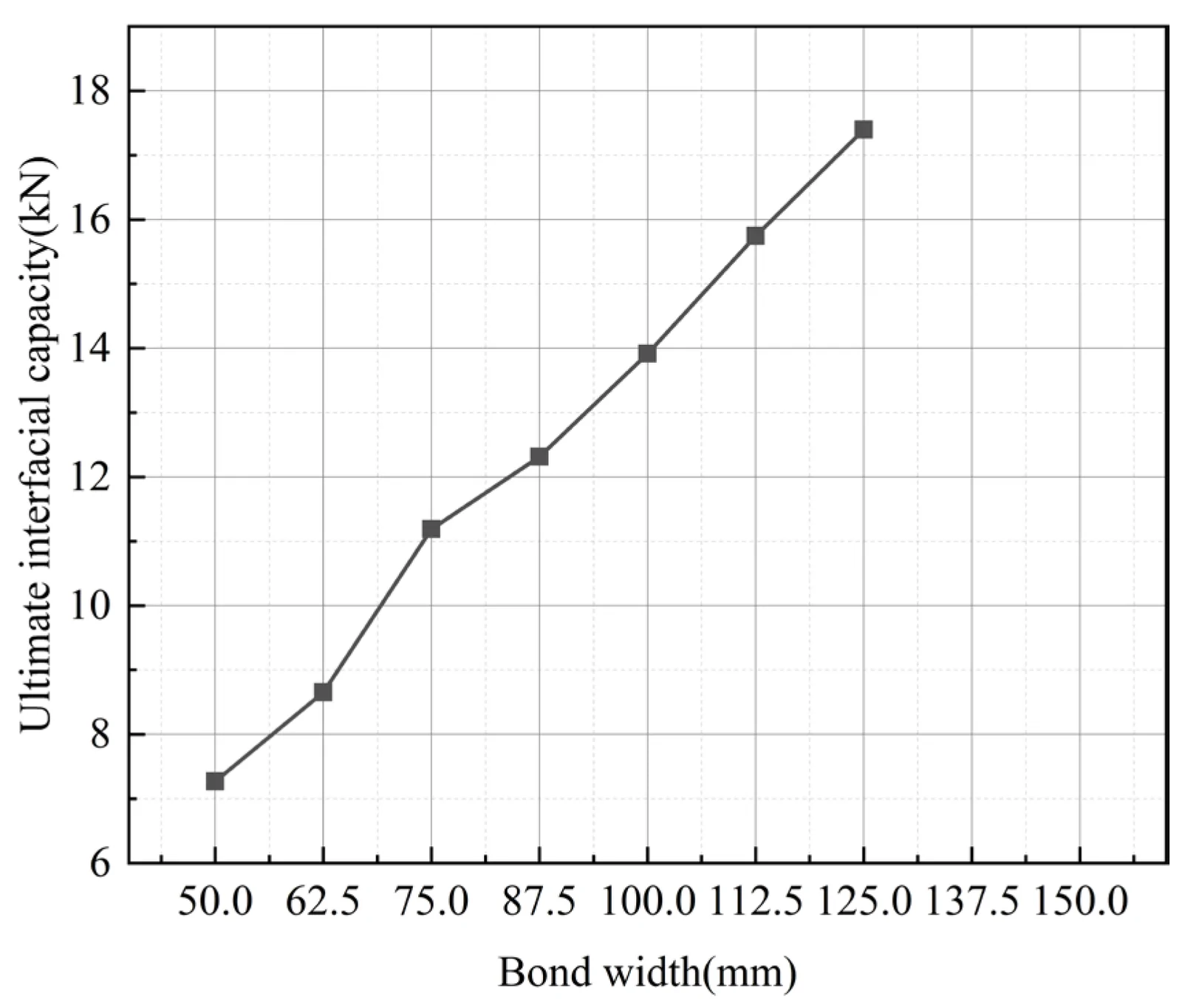
Effect of bond width on ultimate interfacial capacity.

Figure 19 further illustrates the relationship between ultimate interfacial capacity and bond width. As shown in the figure, the ultimate capacity increased monotonically with increasing bond width, with a relatively stable growth rate. When the bond width increased from 50 mm to 125 mm, the ultimate capacity increased from approximately 7.27 kN to 17.40 kN, corresponding to an increase of approximately 139%. This result indicates that bond width is an important geometric parameter affecting the load-carrying performance of CFRP–masonry interfaces within the investigated range.

To avoid evaluating the width effect only based on the absolute peak force, the nominal bond stress listed in Table 4 was used as a normalized index. The nominal bond stress was calculated as the peak shear force transferred by one CFRP–masonry bonded interface divided by the effective bonded area of one interface. The results show that the increase in absolute peak force with bond width does not necessarily correspond to a proportional increase in nominal bond stress. This indicates that the observed increase in capacity is mainly related to the enlarged bonded area and the corresponding extension of the stress-transfer region.

Overall, increasing the bond width enhanced the ultimate interfacial capacity and contributed to maintaining interfacial stiffness during loading within the investigated range. However, the present numerical analysis mainly focused on the influence of bond width under a constant bond length of *L* = 200 mm. The effective bond length was not independently determined in this study. Therefore, the width-related trend should be interpreted within the tested bond-length and bond-width ranges, rather than as a general effective-bond-length rule for CFRP-strengthened masonry interfaces. In addition, existing FRP-to-masonry bond-strength formulations have mostly been developed or calibrated using conventional masonry substrates and adhesive systems. Since the present study focuses on traditional grey brick masonry bonded with glutinous rice mortar, direct application of these formulations may introduce uncertainty. Therefore, the present results are used mainly to interpret the tested material system and geometries, while systematic comparison with existing design formulations should be further examined using expanded experimental datasets.

## 5. Conclusions

This study investigated the interfacial bond behavior between CFRP sheets and traditional glutinous rice mortar-bonded grey brick masonry through experimental and numerical approaches. The influence of CFRP bond width on the interfacial load-carrying performance and load-transfer characteristics was systematically analyzed. The main conclusions are summarized as follows:

(1) The double-shear test results demonstrated that the CFRP–glutinous rice mortar masonry interface exhibited distinct staged mechanical behavior, including the elastic stage, local slip stage, and interfacial failure stage. The dominant failure modes were interfacial debonding between the CFRP sheet and masonry substrate, as well as coupled failure involving interfacial debonding and near-surface masonry damage. These results indicate that interfacial bond behavior plays a critical role in determining the load-transfer capacity of CFRP-strengthened traditional masonry interfaces. CFRP strain measurements indicated that interfacial load transfer gradually developed from the loaded end toward the bonded region, with strain concentration occurring near the loaded end at the peak load stage.
(2) The bond width exhibited a significant influence on the interfacial load-carrying performance. Under different bond length and debonding ratio conditions, increasing the CFRP bond width consistently improved the peak interfacial capacity and initial stiffness. When the bond width increased from 50 mm to 100 mm, the peak capacities increased by 80.3–114.8% under different interface conditions. This enhancement can be attributed to the enlargement of the effective load-transfer region and the reduction in local stress concentration near the loaded end. The reinforcement settings considered in this study included CFRP bond width, bond length, and interface integrity condition. These settings mainly affected the CFRP–masonry interfacial load-transfer capacity and stiffness response, rather than directly modifying the intrinsic flexibility of the brick–mortar joints.
(3) A finite element model was established and used to compare the experimental and numerical force–displacement responses. The numerical results reproduced the overall interfacial mechanical responses observed in the tests. The model was further used to extend the bond width range. For a constant bond length of 200 mm, increasing the bond width from 50 mm to 125 mm increased the ultimate interfacial capacity from 7.27 kN to 17.40 kN, corresponding to an increase of approximately 139%. This increase was mainly related to the enlargement of the effective bonded area and stress-transfer region under the adopted equivalent cohesive-zone model.

Overall, bond width is an important geometric parameter affecting the interfacial bond performance of CFRP-strengthened traditional masonry within the investigated range. Increasing bond width can improve the interfacial load-carrying capacity, mainly through the enlargement of the effective bonded area and stress-transfer region. However, the findings should be interpreted within the tested material system, bond geometries, loading conditions, and laboratory environment. Masonry layout optimization, such as the number of masonry courses or mortar joints per unit area, was not independently investigated in this study. Further studies on durability, material compatibility, environmental exposure, and long-term CFRP–masonry interface behavior are required before the results can be directly extended to broader design applications.

## Figures and Tables

**Table 1:** Compression test results of glutinous rice mortar.

Specimen	Peak Compressive Load (kN)	Compressive Strength (MPa)
M-1	4.48	0.90
M-2	4.46	0.89
M-3	4.43	0.89
M-4	4.44	0.89
M-5	4.59	0.92
M-6	4.60	0.92
Mean	4.50	0.90
SD	0.08	0.015
COV (%)	1.68	1.63

**Table 2:** Physical and compressive properties of fired clay grey bricks.

Specimen	Mass (g)	Density (kg/m^3^)	Peak Compressive Load (kN)	Compressive Strength (MPa)
Brick-1	2262.4	1711	267	20.2
Brick-2	2267.6	1715	266	20.2
Brick-3	2134.1	1614	264	20.0
Brick-4	2378.6	1799	270	20.4
Brick-5	2334.5	1765	253	19.2
Brick-6	2214.7	1675	245	18.5
Mean	2265.3	1713.2	260.8	19.8
SD	83.6	65.3	9.7	0.74
COV (%)	3.69	3.81	3.72	3.76

**Table 3:** Main properties of carbon fiber-reinforced polymer (CFRP) sheet and modified epoxy adhesive.

Material	Property	Value
CFRP sheet	Number of layers	1
CFRP sheet	Nominal thickness	0.111 mm
CFRP sheet	Elastic modulus	2.4 × 10^5^ MPa
CFRP sheet	Tensile strength	3737.7 MPa
CFRP sheet	Rupture strain	1.7%
Modified epoxy adhesive	Adhesive layer thickness	2 mm
Modified epoxy adhesive	Tensile strength	56.3 MPa
Modified epoxy adhesive	Elastic modulus	3.5 × 10^3^ MPa
Modified epoxy adhesive	Elongation at break	2.3%
Modified epoxy adhesive	Flexural strength	81.3 MPa
Modified epoxy adhesive	Compressive strength	89.2 MPa

**Table 4:** Complete specimen matrix and experimental results of double-shear tests.

Configuration	L (mm)	B (mm)	$\eta_{d}$ (%)	$P_{max,1}$ (kN)	$P_{max,2}$ (kN)	Mean $P_{max}$ (kN)	$\tau_{n}$ (MPa)	COV_P_ (%)	$\delta_{u,1}$ (mm)	$\delta_{u,2}$ (mm)	Mean $\delta_{u}$ (mm)	COV_δ_ (%)	Failure Mode
1	100	50	0	4.27	4.03	4.15	0.83	4.00	1.81	1.75	1.78	2.38	ID
2	100	50	10	3.79	4.02	3.91	0.78	4.07	2.11	2.23	2.17	3.91	CD-NSMD
3	100	50	20	3.44	3.29	3.37	0.68	3.26	2.00	1.85	1.93	5.51	ID
4	150	50	0	6.00	6.46	6.23	0.83	5.22	2.78	2.84	2.81	1.51	MD-ID
5	150	50	10	5.09	4.77	4.93	0.66	4.66	2.50	2.38	2.44	3.48	ID
6	150	50	20	4.66	4.90	4.78	0.64	3.48	2.27	2.46	2.37	5.68	ID
7	200	50	0	7.27	6.82	7.05	0.71	4.52	3.86	3.80	3.83	1.11	MD-ID
8	200	50	10	6.65	6.98	6.82	0.68	3.48	3.72	3.97	3.85	4.60	ID
9	200	50	20	5.76	5.45	5.61	0.56	3.91	2.99	2.86	2.93	3.14	ID
10	100	75	0	5.62	5.96	5.79	0.77	4.21	2.78	2.88	2.83	2.50	ID
11	100	75	10	5.26	5.50	5.38	0.72	3.15	2.13	1.96	2.05	5.88	ID
12	100	75	20	4.64	4.95	4.80	0.64	4.57	2.30	2.36	2.33	1.82	CR
13	150	75	0	8.82	8.26	8.54	0.76	4.68	4.02	3.80	3.91	3.98	CR
14	150	75	10	7.77	8.13	7.95	0.71	3.16	2.27	2.43	2.35	4.81	ID
15	150	75	20	6.93	6.53	6.73	0.60	4.15	2.44	2.37	2.41	2.06	ID
16	200	75	0	11.36	12.17	11.77	0.79	4.9	3.82	4.00	3.91	3.26	ID
17	200	75	10	10.14	9.62	9.88	0.66	3.76	3.07	2.81	2.94	6.25	MD-ID
18	200	75	20	8.90	9.33	9.12	0.61	3.3	2.96	3.02	2.99	1.42	MD-ID
19	100	100	0	7.70	7.24	7.47	0.75	4.36	1.80	1.69	1.75	4.46	ID
20	100	100	10	7.46	7.87	7.67	0.77	3.78	1.93	2.01	1.97	2.87	CR
21	100	100	20	6.83	6.53	6.68	0.67	3.18	1.94	1.89	1.92	1.85	ID
22	150	100	0	11.01	11.70	11.36	0.76	4.27	2.45	2.64	2.55	5.28	MD-ID
23	150	100	10	10.46	9.90	10.18	0.68	3.86	2.74	2.64	2.69	2.63	MD-ID
24	150	100	20	9.43	9.89	9.66	0.65	3.37	2.16	2.27	2.22	3.51	ID
25	200	100	0	13.92	13.07	13.50	0.68	4.45	3.42	3.18	3.30	5.14	MD-ID
26	200	100	10	13.00	13.74	13.37	0.67	3.92	2.11	2.17	2.14	1.98	CR
27	200	100	20	12.38	11.80	12.09	0.61	3.39	3.39	3.54	3.47	3.06	CR

Note: ID = interfacial debonding failure; CD-NSMD = coupled failure of CFRP debonding and near-surface masonry damage; CR = CFRP sheet rupture failure; MD-ID = combined masonry damage and interfacial debonding failure; COV = coefficient of variation. The listed failure mode represents the main failure mode observed in the two repeated specimens for each configuration. $P_{max}$ denotes the peak shear force transferred by one bonded interface, calculated as one half of the peak total actuator force recorded by the load cell (HIKWANG, TaiZhou, China); $\tau_{n}$ denotes the nominal bond stress; $\delta_{u}$ denotes the measured relative displacement between the CFRP sheet and the masonry substrate. Subscripts 1 and 2 in $P_{max,1}$, $P_{max,2}$, $\delta_{u,1}$, and $\delta_{u,2}$ denote the two repeated specimens for each configuration.

**Table 5:** Average local interfacial shear stress estimated from CFRP strain gradients for the representative L200 × B100 specimen.

Interval Between Strain Gauges (mm)	${F=0.2P}_{max}$	${F=0.3P}_{max}$	${F=0.5P}_{max}$	${F=0.6P}_{max}$	${F=0.8P}_{max}$	${F=P}_{max}$
10–50	0.260	0.516	0.617	0.944	0.968	1.445
50–90	0.170	0.205	0.377	0.394	1.007	0.455
90–140	0.112	0.171	−0.241	−0.187	0.359	0.744
140–170	0.000	0.047	1.122	1.165	0.416	0.740

**Table 6:** Interface parameters used in the finite element model.

Maximum Equivalent Tangential Contact Stress (MPa)	Critical Fracture Energy of Tangential Separation (J/m^2^)	Maximum Equivalent Normal Contact Stress (MPa)	Critical Fracture Energy of Normal Separation (J/m^2^)	Artificial Damping Coefficient
0.85	800	0.24	68.97	1.0 × 10^−5^

**Table 7:** Quantitative comparison between experimental and numerical results for representative L200 specimens.

Specimen	$P_{exp}$ (kN)	$P_{FE}$ (kN)	Error of $P_{max}$ (%)	$\delta_{exp}$ (mm)	$\delta_{FE}$ (mm)	Error of $\delta_{u}$ (%)	$K_{exp}$ (kN/mm)	$K_{FE}$ (kN/mm)	Error of K (%)
L200 × B50	7.27	7.27	0	3.86	3.86	0	3.41	3.21	5.86
L200 × B75	11.36	11.19	1.50	3.82	3.81	0.26	4.80	4.59	4.38
L200 × B100	13.92	13.92	0	3.42	3.38	1.17	6.71	6.33	5.66

**Table 8:** Extended numerical cases for parametric analysis.

Parameter	Values
Bond length, L	200 mm
Bond width, B	62.5, 87.5, 112.5, and 125 mm

## Data Availability

The original contributions presented in this study are included in the article. Further inquiries can be directed to the corresponding author.

## Abbreviations

The following abbreviations are used in this manuscript:

CFRP	Carbon fiber-reinforced polymer
FRP	Fiber-reinforced polymer
